# Using a novel fuzzy 3-inputs algorithms to control the active hydraulic stabilizer bar with the complex model of the vehicle nonlinear dynamics

**DOI:** 10.1371/journal.pone.0282505

**Published:** 2023-03-07

**Authors:** Tuan Anh Nguyen

**Affiliations:** Faculty of the Mechanical Engineering, Thuyloi University, Hanoi, Vietnam; Hebei University of Technology, CHINA

## Abstract

Under the influence of centrifugal force, the rollover phenomenon may occur. The vehicle rolls over when the wheel is completely separated from the road surface, i.e., the vertical force of the wheel is reduced to zero. To overcome this problem, the active stabilizer bar is used at the front and rear axles of the vehicle. The active stabilizer bar works on the difference in fluid pressure inside the hydraulic motor. This article is aimed at studying the vehicle rollover dynamics when the hydraulic stabilizer bar is used. In this article, the model of a complex dynamic is established. This is a combination of the model of spatial dynamics, the model of nonlinear double-track dynamics, and the nonlinear tire model. The operation of the hydraulic actuator is controlled by a fuzzy algorithm with 3-inputs. The defuzzification rule is determined based on the combination of 27 cases. The process of calculation and simulation is done with four specific cases corresponding to steering angles. In each case, three situations were investigated. Besides, the speed of the vehicle is also gradually increased from v_1_ to v_4_. As a result of the simulation, which was performed in the MATLAB-Simulink environment, the output values such as roll angle, change of the vertical force, and roll index were significantly reduced when the active stabilizer bar was used. If the vehicle does not use the stabilizer bar, the vehicle may roll over in both the second, third, and fourth cases. If the vehicle uses a mechanical stabilizer bar, this also occurs in the third and fourth cases (only at a very high velocity, v_4_). However, the rollover phenomenon did not occur if the vehicle used a hydraulic stabilizer bar controlled by the fuzzy 3-inputs algorithm. In all investigated cases, the stability and safety of the vehicle are always guaranteed. Besides, the responsiveness of the controller is also very good. An experimental process needs to be conducted to verify the correctness of this research.

## 1. Introduction

Today, vehicles can travel at very high speeds. Therefore, rollover accidents often happen. These accidents often have extremely serious consequences for passengers and cargo [1, 2]. The phenomenon of vehicle rollover occurs when the driver suddenly steers at high speed [3]. At this time, a centrifugal force of great magnitude will appear. This force increases in proportion to the centrifugal acceleration. If the speed of movement is increased, or the steering angle is larger, the centrifugal force will increase very quickly [4]. Centrifugal force will generate the rollover moment, which causes the vehicle to roll over. The greater the distance between the vehicle’s center of gravity and the rollover axis *h*_*x*_, the greater the chance of a rollover occurring [5]. Therefore, bulky vehicles are often easier to roll over than small vehicles [6, 7]. In addition, weather and road surface conditions can also influence this phenomenon [8–12]. Under the influence of centrifugal force, the vehicle body will be tilted. Thus, a difference in vertical forces at the two wheels of the same axle will appear. If the roll angle is larger, the difference will also be larger. Once the roll angle of the vehicle reaches the limited value ϕ_max_, that is, the vertical force of the wheel will reach the minimum value *F*_*z*_ = 0, and the rollover phenomenon will occur [13, 14]. With the same steering angle, if the vehicle can roll, the higher the speed, the lower the limited roll angle. This has been proved by Tuan and Thang [15].


$$SSF=\frac{a_{ymax}}{g}=\frac{t_{w}}{h_{x}}$$ (1)

To warn of the limitation of the rollover phenomenon, the roll index (RI) was used. According to Ataei, et al., the static rollover index (SSF) is used when the vehicle is parked on a ramp [16]. This index is determined by the Formula (1). If the center of gravity is decreased and the track width is increased, the value of the SSF will decrease. However, this indicator has no meaning for the problem of rolling over when moving. Tian, et al. introduced the dynamic roll index concept in [17]. This indicator depends on the difference in vertical force at the wheels. If the vehicle rollovers, the value of this indicator will be the maximum, RI = 1 [18].


$$RI=|\frac{F_{z2}-F_{z1}}{F_{z2}+F_{z1}}|$$ (2)

Several solutions have been proposed to limit the rollover phenomenon. In the first solution, the dimensions and weight of the vehicle need to be optimally calculated. This can be simply understood as reducing the height of the center of gravity, increasing the track width, reducing the mass, etc. However, this can affect the maneuverability and transportability of the vehicle. Usually, the fixed parameters of the vehicle, such as weight, size, etc., have been selected very carefully. The second solution is to use some mechatronics systems, such as air suspension systems [19], active suspension systems [20], electronic steering systems [21, 22], electronic brake systems [23], electronic power distribution systems [24], etc. [25–27]. However, the cost of these electronic control systems is quite high. Besides, the effectiveness in ensuring anti-rolling is still not guaranteed. Today, the most effective solution often used is to equip the vehicle with a stabilizer bar. Unlike the systems outlined above, the stabilizer bar focuses solely on balancing the load between the two sides of the wheel. Therefore, its efficiency is higher [28].

Currently, there are three types of stabilizer bars used in automobiles. First, a mechanical stabilizer bar (also known as a passive stabilizer bar) has a simple structure and is compact. This bar is made from steel with a circular cross-section and is hollow inside. The swingarm of the stabilizer bar is attached to the wheel hub (unsprung mass), and the back of the bar is attached to the chassis via two bearings [29]. The cost of the passive stabilizer bar is quite low, with long service life. Therefore, it is equipped with the most popular vehicles. However, the impact force that is generated by the mechanical stabilizer bar is quite small. Therefore, its safety performance is still not good. Therefore, some models have used hydraulic stabilizer bars to replace conventional passive stabilizer bars. The hydraulic stabilizer bar consists of a hydraulic motor, hydraulic pump, and fluid lines. The difference in pressure inside the hydraulic motor will create an impact force on the two lever arms of the rod. The impact force of the hydraulic stabilizer bar is much larger than that of the mechanical stabilizer bar. Therefore, its efficiency is higher. Besides, an electronic stabilizer bar has also been used on some high-end vehicles. The sensitivity of the electronic stabilizer bar is very high. However, the issue of cost has not yet been resolved. Both the hydraulic stabilizer bar and the electronic stabilizer bar are referred to as the "active stabilizer bar" [30].

Many studies on active stabilizer bars have been published in recent years. In [31], Muniandy, et al. designed a PI-PD (Proportional Integral-Proportional Derivative) controller for an active stabilizer bar. The controller parameters are tuned by the fuzzy algorithm. This problem was also taken up by Dawei, et al. [32]. According to Zhuo, et al., the roll angle of the vehicle body can be reduced if the active stabilizer bar is used to replace the conventional passive stabilizer bar. This is confirmed under the same conditions of lateral acceleration a_y_ [33]. The above studies have not mentioned the influence of the actuator when simulating a vehicle’s oscillation. In [34], Nguyen published an article on the control problem for the active stabilizer bar. In this article, he clearly describes the interaction of the hydraulic actuator. In [35], Dawei, et al. used an experimental method to verify the effectiveness of the active stabilizer bar. This model consists of two hydraulic cylinders, which are mounted at either end of the active stabilizer bar. The force sensor will be located at the bearing position. For the system to work stably, the control signal needs to change continuously based on the excitation signal. Therefore, Nguyen used a fuzzy algorithm to control the hydraulic actuator. In [36], this algorithm includes only a single input signal. Therefore, the response time of the system is still not good. This algorithm can be improved further if there are two input signals [37]. Based on the results obtained, the active stabilizer bar can provide better roll control than the passive stabilizer bar [38–40].

Based on the foregoing, this article focuses on evaluating the efficiency of the hydraulic stabilizer bar. Compared with previous studies, this article uses a complex dynamic model. This is a combination of the model of spatial dynamics and the model of nonlinear dynamics. In addition, the fuzzy 3-inputs algorithm is designed to control the operation of the hydraulic actuator. This is the new content that is introduced in the article. The content of this article consists of four sections. In the first section, some introductions and reviews of the stabilizer bar are made. In the second section, the complex dynamics model and control algorithm are established. Then the simulation is done. These results are analyzed and evaluated in detail in the third section. Finally, some conclusions are indicated in the fourth section.

## 2. Methodology

### 2.1 Model of the vehicle dynamics

A dynamics model needs to be established to simulate the vehicle’s oscillations when steering. A complete dynamics model will include an oscillation model, a motion model, and a tire model. Most of the previous studies only used the half-dynamics model and the linear single-track dynamics model. Besides, the linear tire model is also integrated. The model is quite simple; however, many of the effects of oscillation have been overlooked. Therefore, the accuracy is not high. In this article, the author will establish complex dynamics models, including the model of spatial dynamics with 7 degrees of freedom, the model of nonlinear double-track dynamics with 3 degrees of freedom, and the nonlinear tire model. This is a new combination.

**Fig 1:**
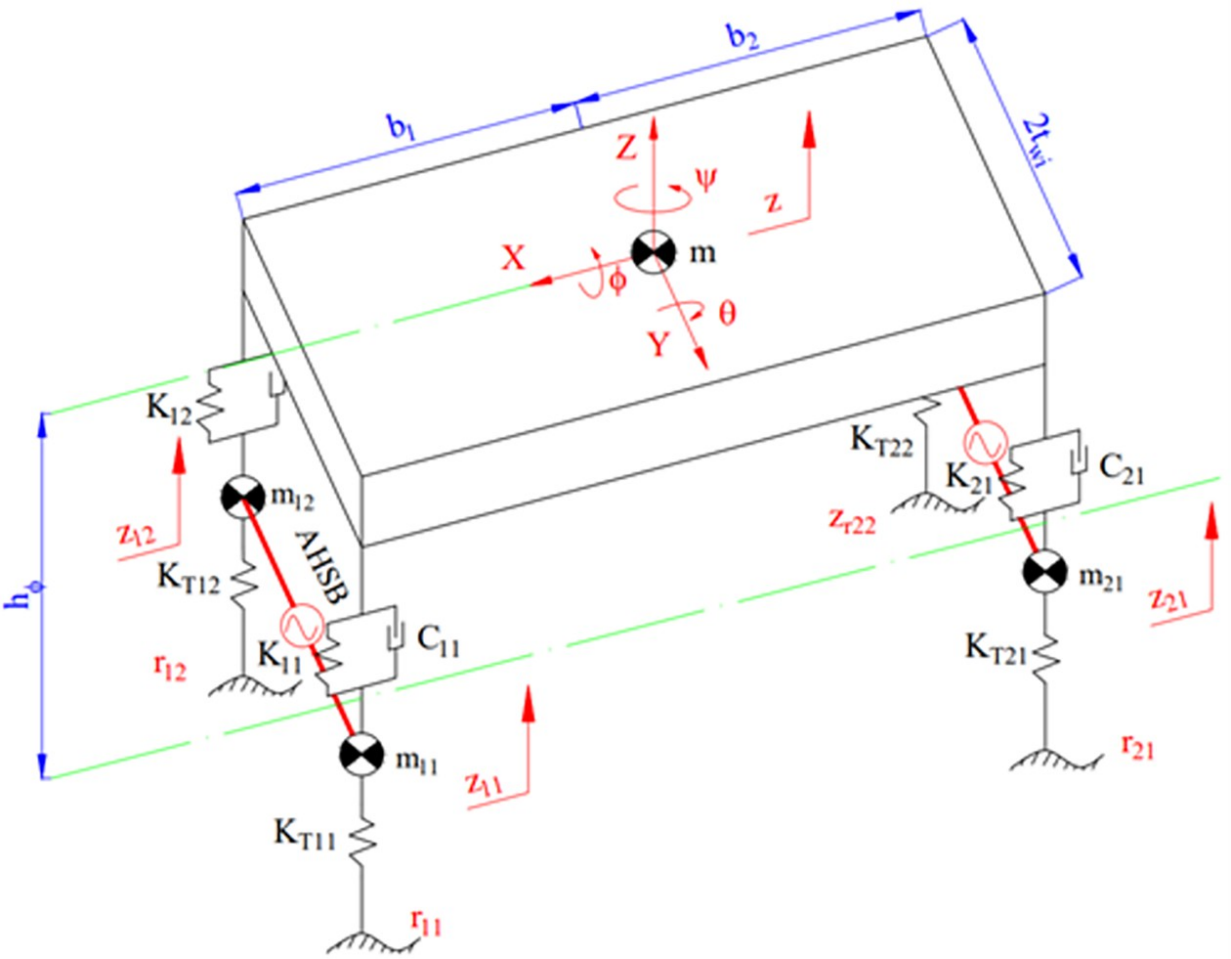
Model of the spatial dynamics.

A spatial dynamics model is shown in Fig 1. This model consists of one sprung and four unsprung masses. The vehicle body (sprung mass) performs three displacements, while each unsprung mass performs only one. Using the separation method, a car model can be separated into five objects having seven degrees of freedom (DOFs). Therefore, this model is also known as the "full dynamics model" (7 DOFs model). With the model of the spatial dynamics in Fig 1, the equations describing the vehicle’s oscillation are given as follows:

$$F_{K11}+F_{C11}+F_{K12}+F_{C12}+F_{K21}+F_{C21}+F_{K22}+F_{C22}-F_{im}=0$$ (3)

$$(F_{K11}+F_{C11}-F_{K12}-F_{C12})t_{w1}+(F_{K21}+F_{C21}-F_{K22}-F_{C22})t_{w2}-M_{\phi }=0$$ (4)

$$(F_{K11}+F_{C11}+F_{K12}+F_{C12})b_{1}-(F_{K21}+F_{C21}+F_{K22}+F_{C22})b_{2}-M_{\theta }=0$$ (5)

$$F_{KT11}-F_{K11}-F_{C11}-F_{AHSB11}-F_{i11}=0$$ (6)

$$F_{KT12}-F_{K12}-F_{C12}+F_{AHSB12}-F_{i12}=0$$ (7)

$$F_{KT21}-F_{K21}-F_{C21}-F_{AHSB21}-F_{i21}=0$$ (8)

$$F_{KT22}-F_{K22}-F_{C22}+F_{AHSB22}-F_{i22}=0$$ (9)

Where:

$$F_{im}=m\ddot{z}$$ (10)

$$F_{i11}=m_{11}{\ddot{z}}_{11}$$ (11)

$$F_{i12}=m_{12}{\ddot{z}}_{12}$$ (12)

$$F_{i21}=m_{21}{\ddot{z}}_{21}$$ (13)

$$F_{i22}=m_{22}{\ddot{z}}_{22}$$ (14)

$$M_{\phi }=(J_{x}+mh_{x}^{2})\ddot{\phi }$$ (15)

$$M_{\theta }=(J_{y}+mh_{y}^{2})\ddot{\theta }$$ (16)

Eqs from (3) to (9) can be rewritten in reduced form as follows:

$$m\ddot{z}=\sum_{i,j=1}^{2}\left(F_{Kij}+F_{Cij}\right)$$ (17)

$$(J_{x}+mh_{x}^{2})\ddot{\phi }=\sum_{i,j=1}^{2}\left[{\left(-1\right)}^{j-1}(F_{Kij}+F_{Cij})t_{wi}\right]+\{gsin\phi +[{\dot{v}}_{y}+(\dot{\beta }+\dot{\psi })v_{x}]cos\phi \}mh_{x}$$ (18)

$$(J_{y}+mh_{y}^{2})\ddot{\theta }=\sum_{i,j=1}^{2}{\left(-1\right)}^{i-1}(F_{Kij}+F_{Cij})b_{i}$$ (19)

$$m_{ij}{\ddot{z}}_{ij}=F_{KTij}-F_{Kij}-F_{Cij}+{\left(-1\right)}^{j}F_{AHSBij}$$ (20)

Where:

$$F_{AHSB11}=F_{AHSB12}$$ (21)

$$F_{AHSB21}=F_{AHSB22}$$ (22)

**Fig 2:**
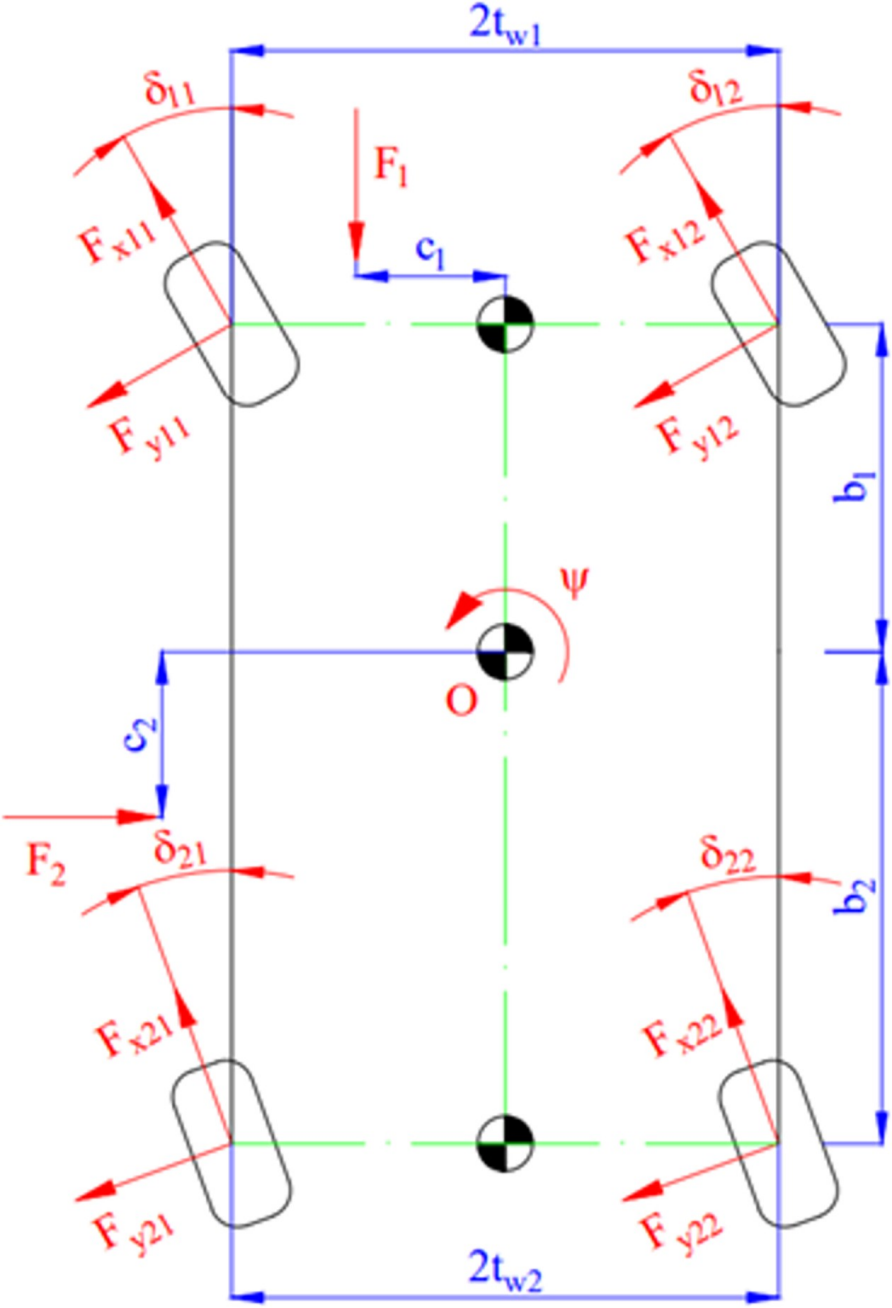
Model of the nonlinear double-track dynamics.

The components of longitudinal velocity *v*_*x*_, lateral velocity *v*_*y*_, and yaw angle *ψ* are determined through a nonlinear double-track dynamics model (Fig 2). This model includes three degrees of freedom, which correspond to the three directions of motion of the car. This is a complex nonlinear model that considers the tires’ deformation during travel.


$$\begin{array}{l} F_{x11}cos\delta_{11}-F_{y11}sin\delta_{11}+F_{x12}cos\delta_{12}-F_{y12}sin\delta_{12}+F_{x21}cos\delta_{21}\\ -F_{y21}sin\delta_{21}+F_{x22}cos\delta_{22}-F_{y22}sin\delta_{22}-F_{1}-F_{ix}+F_{cex}=0 \end{array}$$ (23)

$$\begin{array}{l} F_{x11}sin\delta_{11}+F_{y11}cos\delta_{11}+F_{x12}sin\delta_{12}+F_{y12}cos\delta_{12}+F_{x21}sin\delta_{21}\\ +F_{y21}cos\delta_{21}+F_{x22}sin\delta_{22}+F_{y22}cos\delta_{22}-F_{2}-F_{iy}-F_{cey}=0 \end{array}$$ (24)

$$\begin{array}{l} (F_{x11}sin\delta_{11}+F_{y11}cos\delta_{11})b_{1}-(F_{x11}cos\delta_{11}-F_{y11}sin\delta_{11})t_{w1}+(F_{x12}sin\delta_{12}+F_{y12}cos\delta_{12})b_{1}\\ +(F_{x11}cos\delta_{11}-F_{y11}sin\delta_{11})t_{w1}-(F_{x21}sin\delta_{21}+F_{y21}cos\delta_{21})b_{2}-(F_{x21}cos\delta_{21}-F_{y21}sin\delta_{21})t_{w2}\\ -(F_{x22}sin\delta_{22}+F_{y22}cos\delta_{22})b_{2}+(F_{x22}cos\delta_{22}-F_{y22}sin\delta_{22})t_{w2}\\ -F_{1}c_{1}-F_{2}c_{2}-M_{z11}-M_{z12}-M_{z21}-M_{z22}-M_{\psi }=0 \end{array}$$ (25)

Where:

$$F_{ix}=(m+m_{11}+m_{12}+m_{21}+m_{22}){\dot{v}}_{x}$$ (26)

$$F_{iy}=(m+m_{11}+m_{12}+m_{21}+m_{22}){\dot{v}}_{y}$$ (27)

$$F_{cex}=(m+m_{11}+m_{12}+m_{21}+m_{22})(\dot{\beta }+\dot{\psi })v_{y}$$ (28)

$$F_{cey}=(m+m_{11}+m_{12}+m_{21}+m_{22})(\dot{\beta }+\dot{\psi })v_{x}$$ (29)

$$M_{\psi }=J_{z}\ddot{\psi }$$ (30)

Eqs (23), (24), and (25) can be rewritten in reduced form as follows:

$$\sum_{i,j=1}^{2}\left(m+m_{ij}\right)[{\dot{v}}_{x}-(\dot{\beta }+\dot{\psi })v_{y}]=\sum_{i,j=1}^{2}\left(F_{xij}cos\delta_{ij}-F_{yij}sin\delta_{ij}\right)-F_{1}$$ (31)

$$\sum_{i,j=1}^{2}\left(m+m_{ij}\right)[{\dot{v}}_{y}+(\dot{\beta }+\dot{\psi })v_{x}]=\sum_{i,j=1}^{2}\left(F_{xij}sin\delta_{ij}+F_{yij}cos\delta_{ij}\right)-F_{2}$$ (32)

$$J_{z}\ddot{\psi }=\sum_{i,j=1}^{2}\left[{\left(-1\right)}^{j}(F_{xij}cos\delta_{ij}-F_{yij}sin\delta_{ij})t_{wi}+{\left(-1\right)}^{i+1}(F_{xij}sin\delta_{ij}+F_{yij}cos\delta_{ij})b_{i}+F_{i}c_{i}-M_{zij}\right]$$ (33)

The forces and moments at the wheel can be calculated according to the tire model. The linear tire model is the simplest; however, the calculation error is very high. Tire models such as Burckhardt, Ammon, Dugoff, HSRI (Highway Safety Research Institute), etc. can be used to determine these values [41, 42]. In particular, the Pacejka tire model has very high accuracy. However, this model is quite complex. In this article, the Pacejka tire model with experimental parameters is used [43]. The values of force and moment are functions of the slip ratio, slip angle, vertical force of the wheel, etc.


$$F_{x}=D_{x1}sin\{D_{x2}arctan[D_{x3}(1-D_{x4})(s+D_{x5})+D_{x4}arctan(D_{x2}(s+D_{x5}))]\}+D_{x6}$$ (34)

$$F_{y}=D_{y1}sin\{D_{y2}arctan[D_{y3}(1-D_{y4})(\alpha +D_{y5})+D_{y4}arctan(D_{y2}(\alpha +D_{y5}))]\}+D_{y6}$$ (35)

$$F_{y}=D_{z1}sin\{D_{z2}arctan[D_{z3}(1-D_{z4})(\alpha +D_{z5})+D_{z4}arctan(D_{z2}(\alpha +D_{z5}))]\}+D_{z6}$$ (36)

Where: *D*_*xi*_, *D*_*yi*_, *D*_*zi*_ are the coefficients of the model; *s* is the slip ratio; *α* is the slip angle.

The impact force generated at the arm of the stabilizer bar is generated by the differential pressure inside the hydraulic motor. When current is supplied, the valves inside the motor move. This movement created a pressure difference between the compartments. So, the motor will work. The operating principle of the hydraulic actuator is shown by the following equations.


$${\dot{X}}_{sv}\tau +X_{sv}-K_{sv}u(t)=0$$ (37)

$$D_{m}{\dot{\varphi }}_{m}+K_{ce}\Delta P+\frac{V_{t}}{4\beta_{e}}\Delta \dot{P}-K_{qi}X_{sv}=0$$ (38)

$$J_{m}{\ddot{\varphi }}_{m}+B_{m}{\dot{\varphi }}_{m}+T_{r}-D_{m}\Delta P=0$$ (39)

In the case of cars using only mechanical stabilizer bars, the force generated by the stabilizer bar is calculated by Formula (40), according to [29]. This force is a function of the displacement of the unsprung mass. The symbols *a*, *b*, *c*, *d*, *EJ*_*x*_, and *GJ*_*p*_ can be referenced in [29].


$$z_{ij}=\frac{F_{MSB}{\left(d+b\right)}^{3}}{3EJ_{x}}+ctan\int_{0}^{a+b}\frac{F_{MSB}c}{GJ_{p}}dz$$ (40)

### 2.2 Fuzzy 3-inputs control algorithm

**Fig 3:**
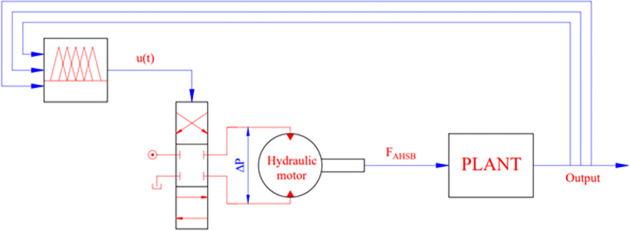
Schematic of the control system.

**Fig 4:**
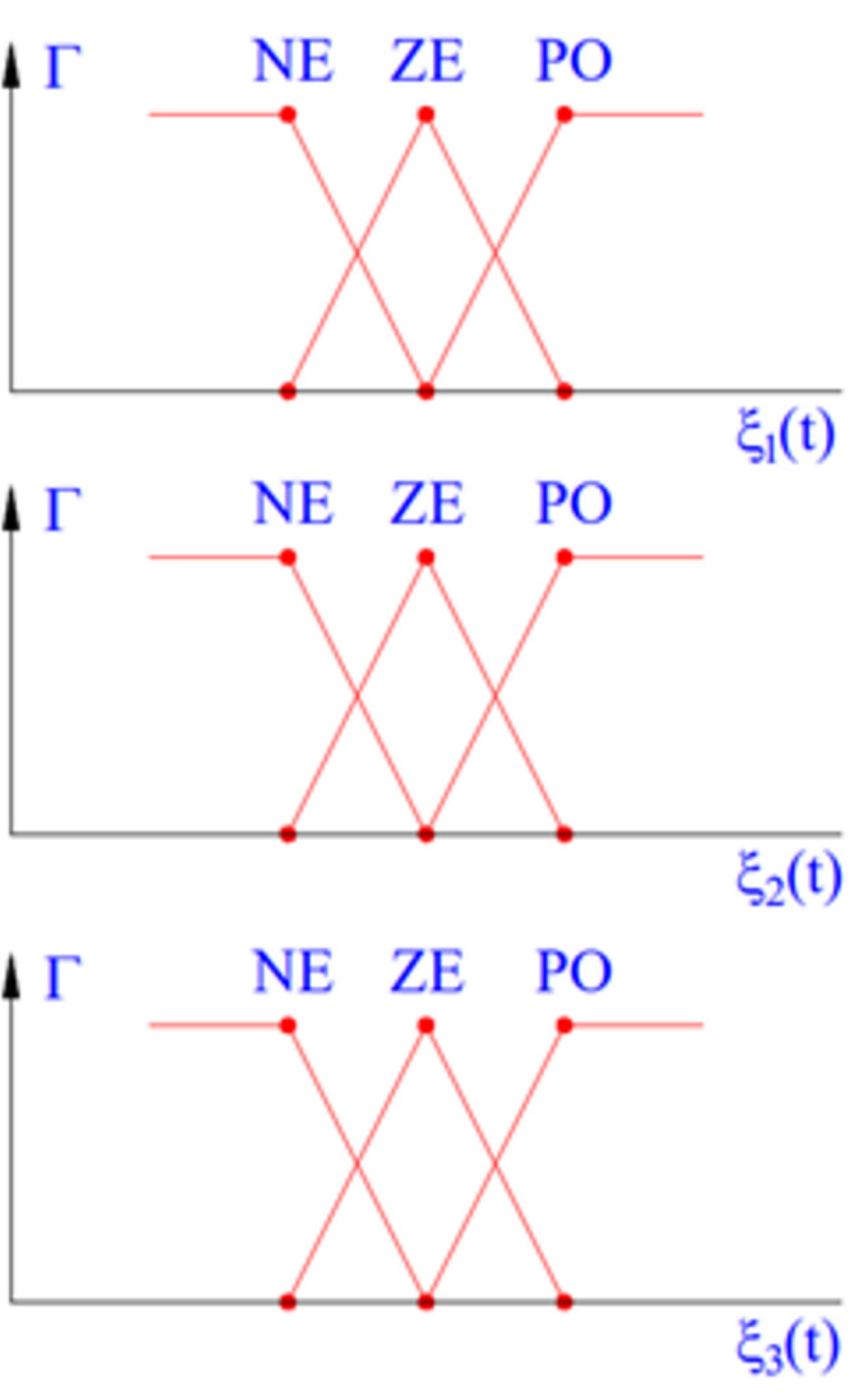
Membership function.

The performance of the system depends on the algorithm designed for the controller. In this article, a fuzzy control algorithm with 3-inputs is used. A schematic diagram of the system is shown in Fig 3. The controller inputs include roll angle, displacement of the unsprung mass, and the change of the vertical force at the wheels. The relationship between the elements is determined by the membership function (Fig 4). This membership function is described by the Formula (41):

$$\Gamma (x,\xi )=\{\begin{array}{l} 1,x<\xi_{a}\\ \frac{x-a}{b-a},\xi_{a}\leq x\leq \xi_{b}\\ \frac{c-x}{c-b},\xi_{b}\leq x\leq \xi_{c}\\ 1,x>\xi_{c} \end{array}=max(min(\frac{x-a}{b-a},\frac{c-x}{c-b}),0)$$ (41)

**Fig 5:**
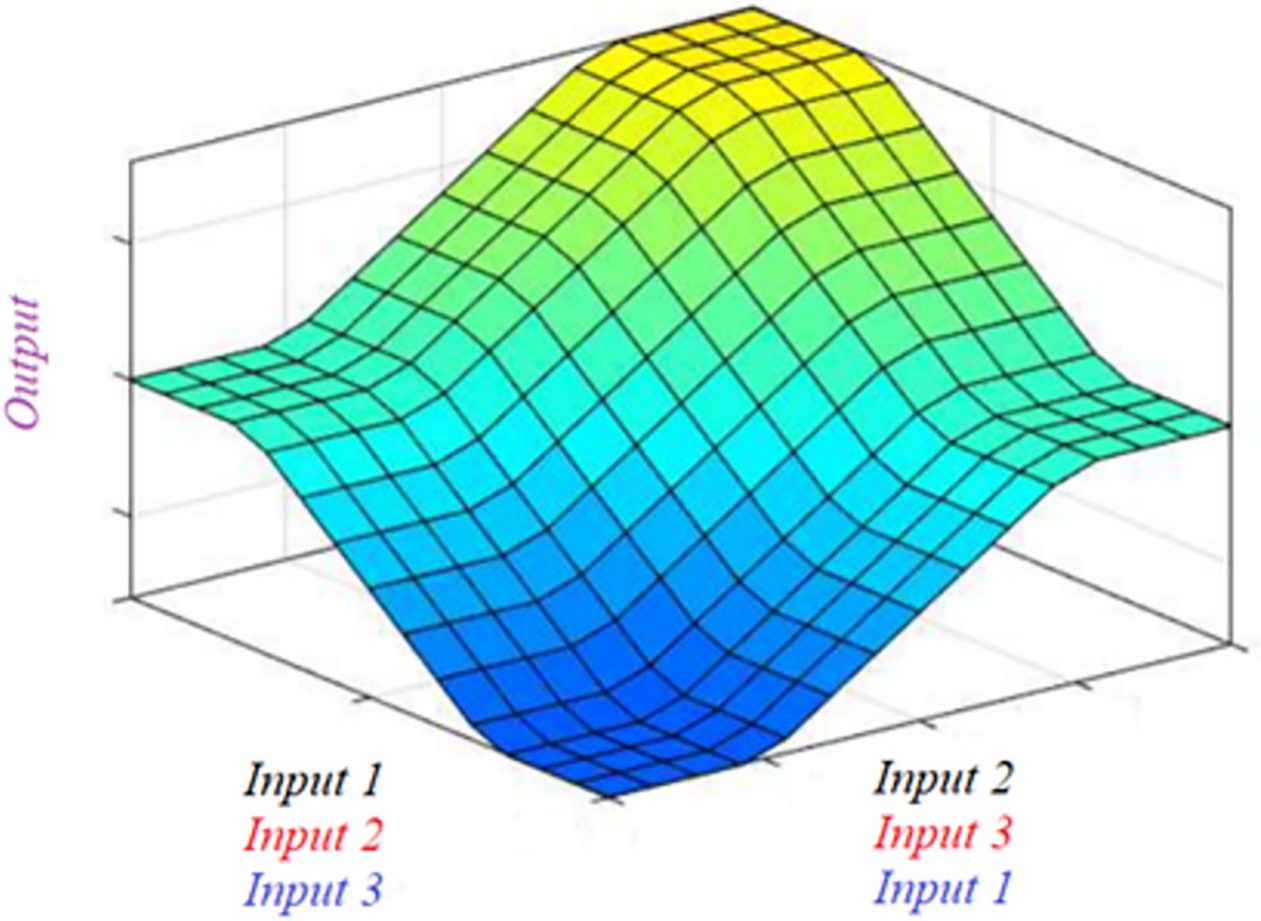
Rules surface.

The defuzzification process is performed based on the fuzzy rule, which has been designed for the controller. Choosing fuzzy rules for the controller is extremely difficult. There are several methods to choose fuzzy rules, such as using optimization algorithms, using repeated simulations to find the optimal value, doing experiments, etc. Each researcher has different views on choosing fuzzy rules for their research. In this article, the author proposes a fuzzy rule with three inputs that are established based on three specific views related to vehicle rollover. From the first point of view, the vehicle roll angle should be small when the vehicle is steering. Although the roll angle can be reduced, the displacement of the unsprung mass can still be significant if the vehicle body is tilted. Therefore, reducing the displacement of the unsprung mass is the second point of view. The wheel is completely lifted off the road when the change in dynamic force is too significant, i.e., the vertical force at the wheel approaches zero. Therefore, it is necessary to reduce the change of dynamic force at the wheel to help the wheel better interact with the road surface; this is the third point of view. Based on these three views, the author proposes a fuzzy rule that uses all three values above as input parameters for the controller. In the author’s opinion, the fuzzy rule is a combination of three elements with 27 cases. This content is presented in Table 1 and Fig 5.

**Table 1:** Fuzzy rules.

Input 1	Input 2	Input 3	Output
NE	NE	NE	VN
NE	NE	ZE	BN
NE	NE	PO	SN
NE	ZE	NE	BN
NE	ZE	ZE	SN
NE	ZE	PO	ZE
NE	PO	NE	SN
NE	PO	ZE	ZE
NE	PO	PO	SP
ZE	NE	NE	BN
ZE	NE	ZE	SN
ZE	NE	PO	ZE
ZE	ZE	NE	SN
ZE	ZE	ZE	ZE
ZE	ZE	PO	SP
ZE	PO	NE	ZE
ZE	PO	ZE	SP
ZE	PO	PO	BP
PO	NE	NE	SN
PO	NE	ZE	ZE
PO	NE	PO	SP
PO	ZE	NE	ZE
PO	ZE	ZE	SP
PO	ZE	PO	BP
PO	PO	NE	SP
PO	PO	ZE	BP
PO	PO	PO	VP

After the dynamics model and the controller have been established, the simulation process needs to be conducted with specific cases.

## 3. Simulation and results

### 3.1 Simulation cases

**Fig 6:**
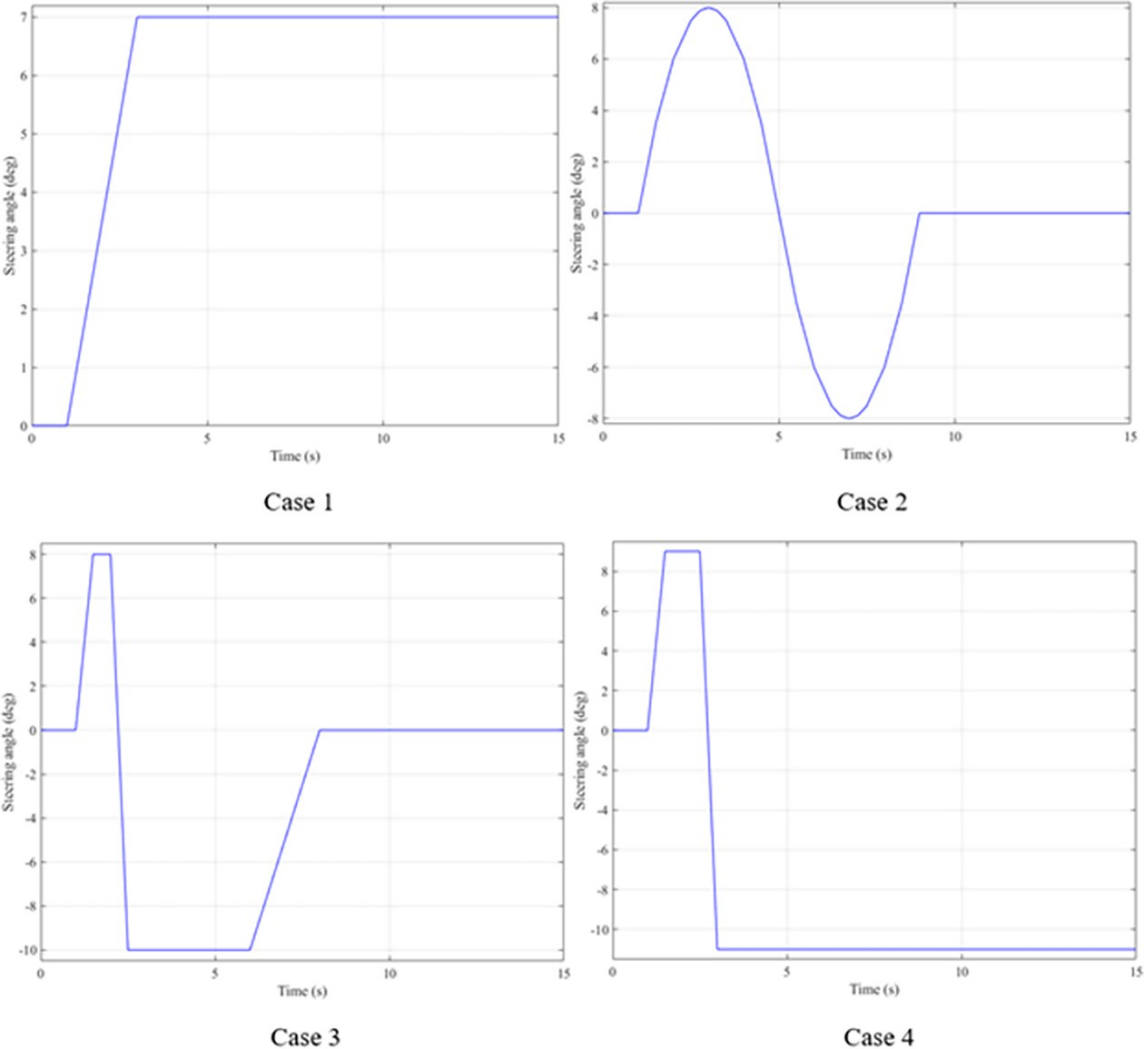
Steering angle.

For rollover studies, there are several commonly used steering angles, such as: "J-turn," "fishhook," "double lane change," etc. In this article, all three types of steering are used. The values of the steering angles are shown in Fig 6.

The parameters used for the simulation are shown in Table 2. Once the inputs have been determined, the simulation can be performed. This process is solved by the software MATLAB-Simulink. There are four cases, corresponding to four steering angles. In each case, the vehicle’s speed will be changed to correspond to four values, including: v_1_ = 70 (km/h)–a; v_2_ = 80 (km/h)–b; v_3_ = 90 (km/h)–c; and v_4_ = 100 (km/h)–d. To evaluate the effectiveness of the active stabilizer bar, the outputs of the problem are compared in three situations: the vehicle using the active stabilizer bar is controlled by the Fuzzy 3-inputs algorithm; the vehicle using the mechanical stabilizer bar, and the vehicle not using a stabilizer bar.

**Table 2:** Specification parameters.

Symbol	Description	Value	Unit
m	Sprung mass	1790	kg
m_ij_	Unsprung mass	49	kg
h_x_	Distance from center of gravity to roll axis	0.52	m
h_y_	Distance from center of gravity to pitch axis	0.50	m
t_wi_	Half of the track width front/rear axle	0.730/0.725	m
b_i_	Distance from center of gravity to front/rear axle	1.20/1.64	m
J_x_	Moment of inertia of the x-axis	670	kgm^2^
J_y_	Moment of inertia of the y-axis	2780	kgm^2^
J_z_	Moment of inertia of the z-axis	2715	kgm^2^
τ	Time constant	0.005	s
K_sv_	Servo valve gain	0.03	m/A
K_qi_	Valve flow gain coefficient	0.02	m^2^/s
K_ce_	Total flow pressure coefficient	4×10^−11^	m^5^/Ns
V_t_	Total volume of trapped oil	1×10^−3^	m^3^
β_e_	Effective bulk modulus of the oil	6×10^6^	N/m^2^
D_m_	Flow per revolution	1.6×10^−5^	m^3^/rad
B_m_	Viscous friction coefficient	10	Nms/rad
J_m_	Moment of inertia of the hydraulic motor	3.5	kgm^2^

### 3.2 Results

The input parameters of the simulation problem include velocity, steering angle, etc. Output values calculated by MATLAB-Simulink software include roll angle, vertical force, and RI. These outcomes are determined based on specific cases and situations.

#### 3.2.1 First case

**Fig 7:**
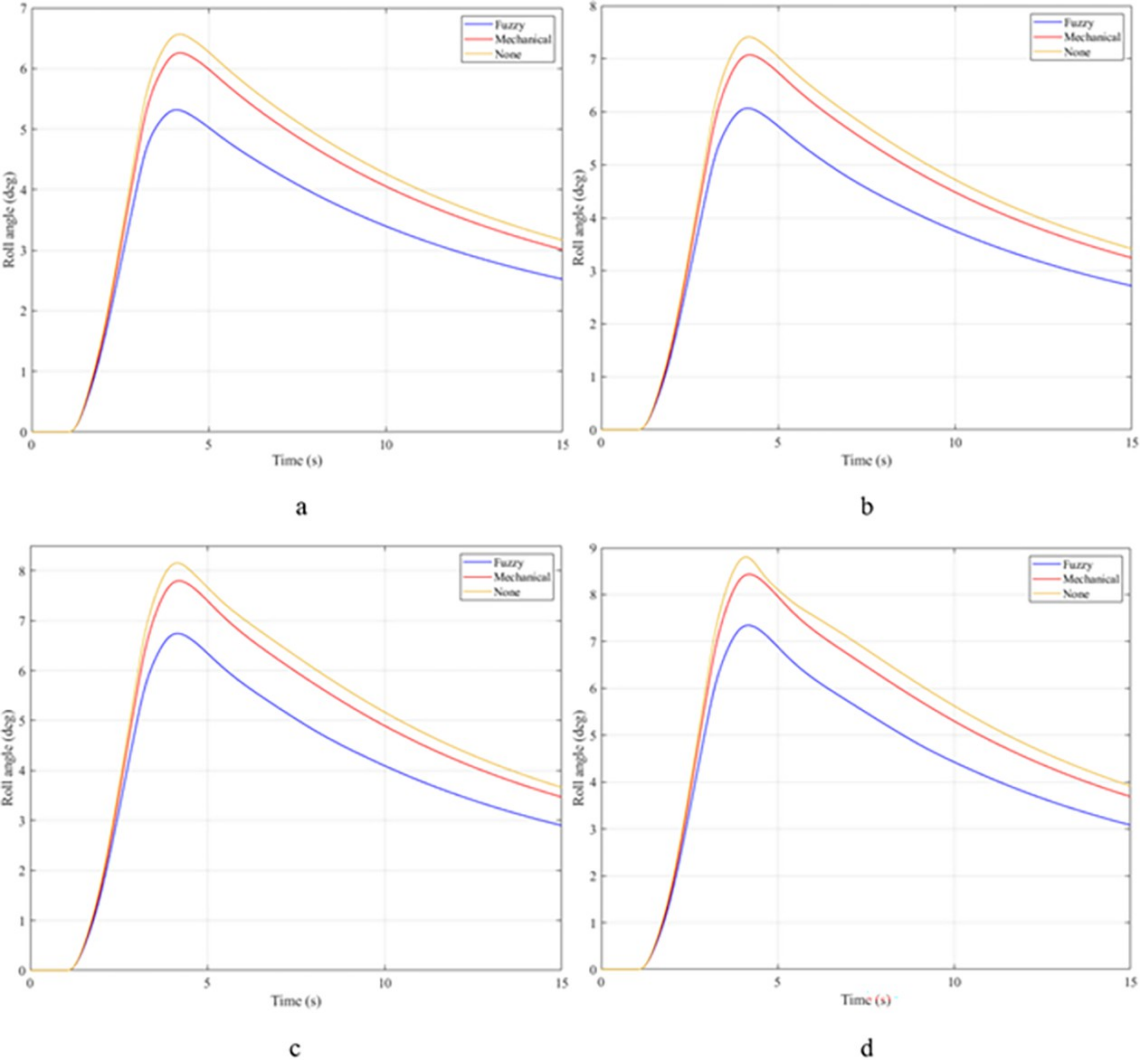
Roll angle.

In the first case, a "J-turn" steering angle is used. The value of the steering angle increases linearly from the first to the third second. The maximum value of the steering angle is 7°. Once the steering angle has reached its maximum value, this value will be maintained for a certain time. The value of the roll angle will increase from zero to the maximum value. This is shown in Fig 7. When the vehicle is moving with a velocity of v_1_ = 70 (km/h), the maximum value of the roll angle can reach 5.32°, 6.26°, and 6.57°, corresponding to three situations: the vehicle uses an active stabilizer bar, the vehicle uses a passive stabilizer bar, and the vehicle does not have a stabilizer bar. Although the value of the steering angle has been constant since t = 3 (s), the value of the roll angle still tends to decrease. The cause of this phenomenon is the nonlinear deformation of the tire. The lateral force on the tire will gradually decrease, so the centrifugal force will also gradually decrease. This causes the vehicle’s roll angle to decrease over time. However, the value of the roll angle cannot be zero if the steering angle persists. As the vehicle’s speed increases, the value of the roll angle also increases rapidly. At a velocity of v_4_ = 100 (km/h), the difference in roll angle values in the three investigated situations has changed greatly. If the vehicle does not have a stabilizer bar, the roll angle of the vehicle can be up to 8.80°. In contrast, this value can be drastically reduced, down to only 7.35° if the vehicle uses an active stabilizer bar.

**Fig 8:**
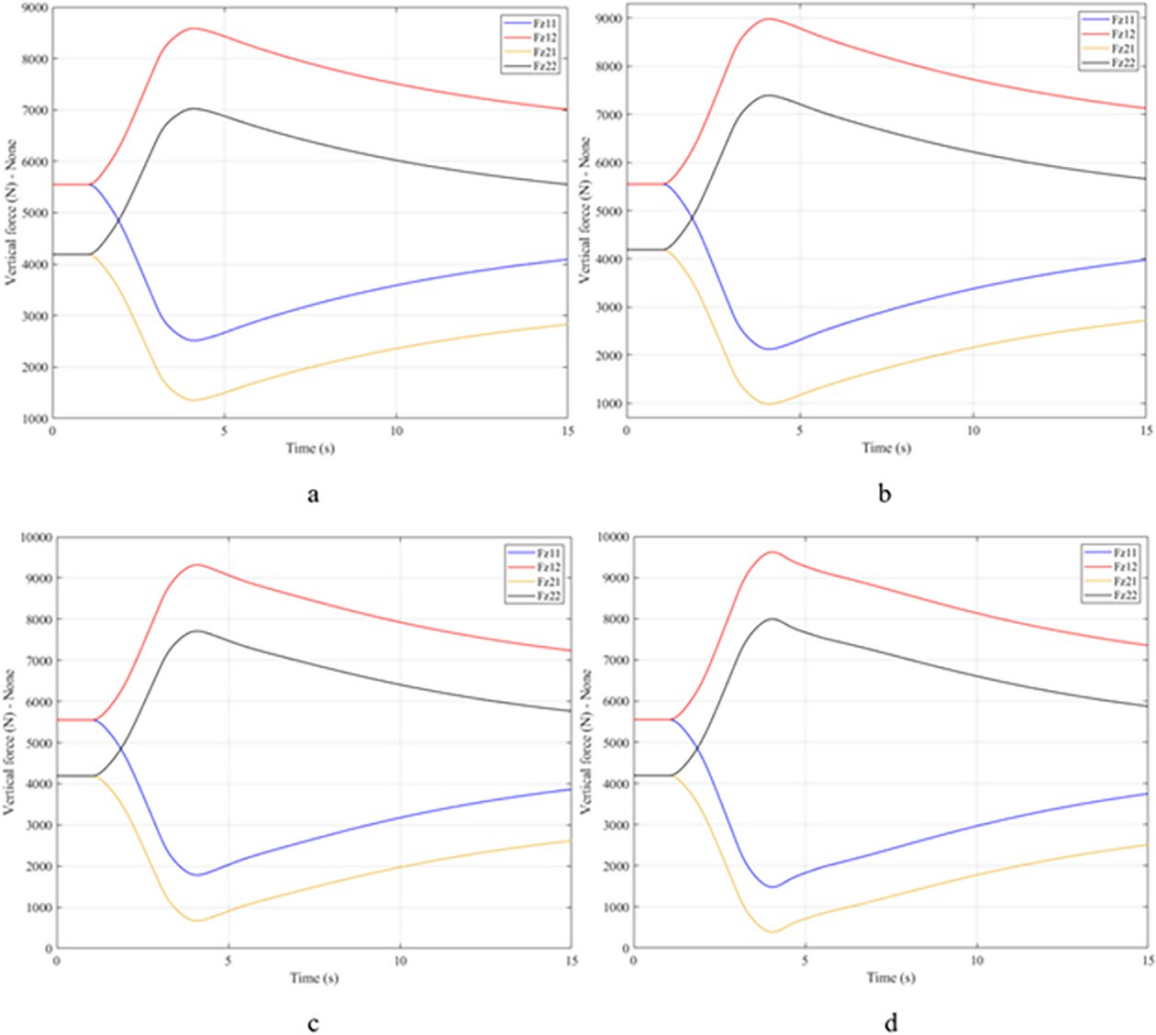
Vertical force–None.

**Fig 9:**
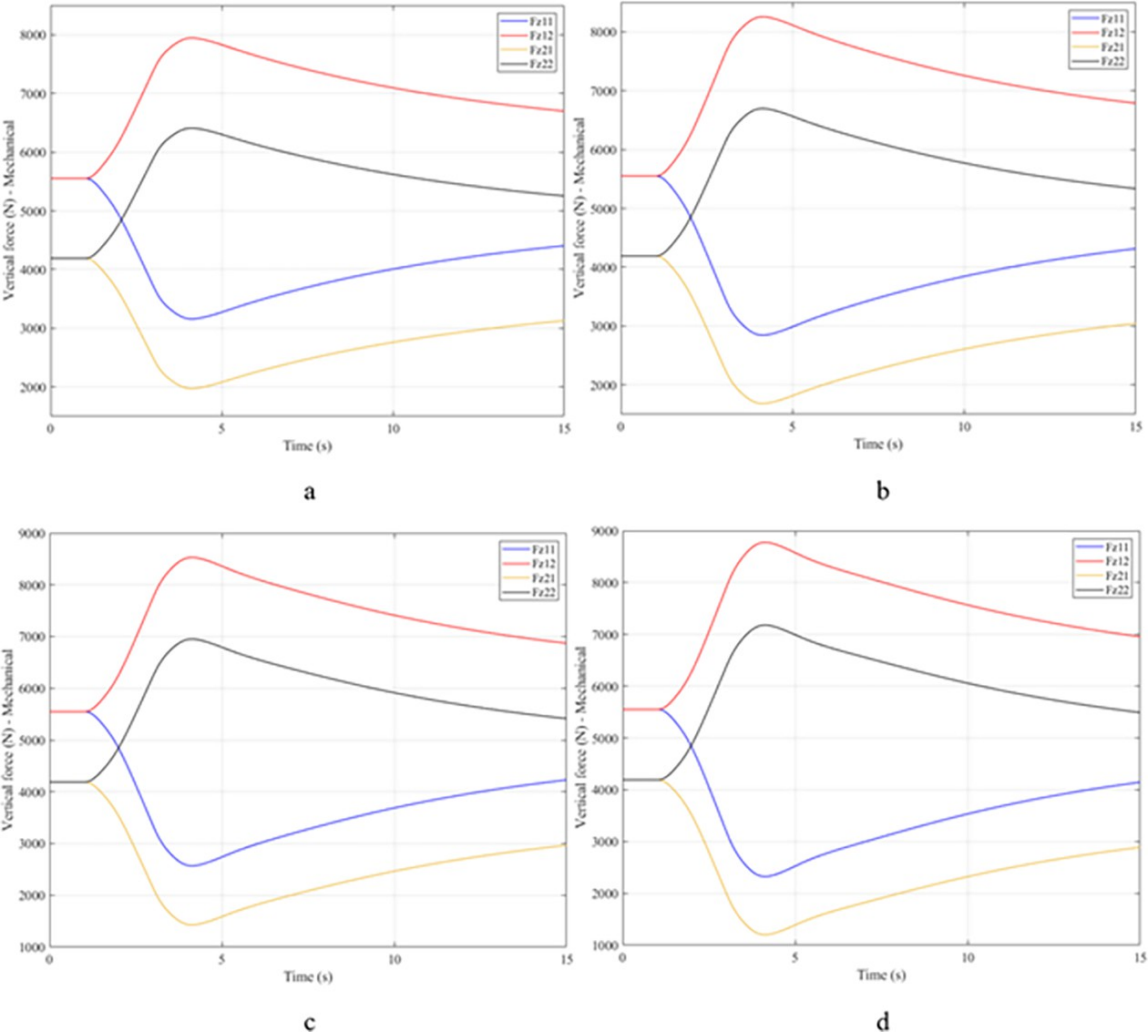
Vertical force–Mechanical.

**Fig 10:**
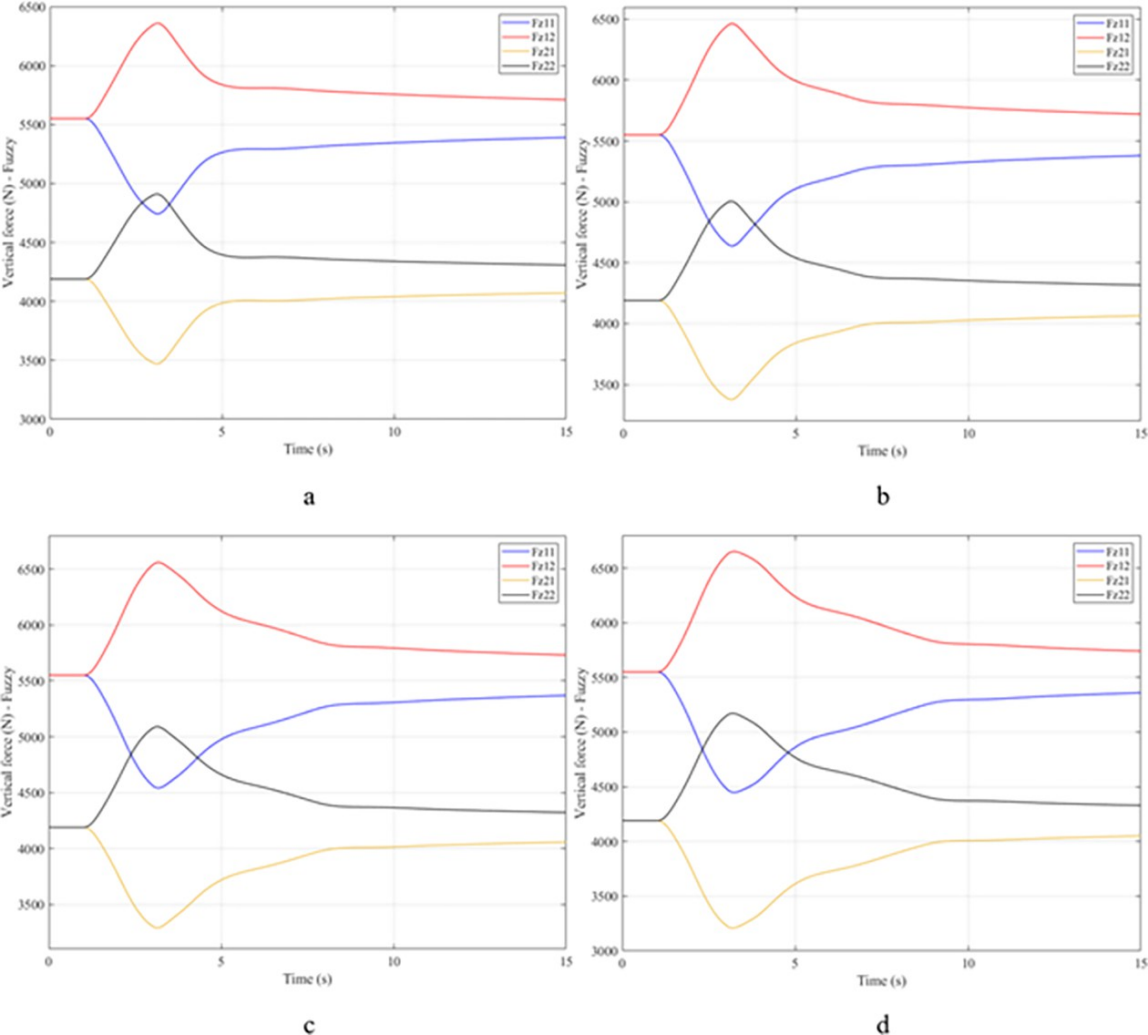
Vertical force–Fuzzy.

When the vehicle body is tilted, a difference in vertical force at the wheels will also appear. For two wheels on either side of the same axle, one side will receive the extra load, and the other side will reduce the load. If the load on the wheel is reduced drastically, the contact between the wheel and the road will also be greatly affected. Once these values approach zero, the wheel may become detached from the road surface. At this time, the rollover phenomenon can happen unexpectedly. The variation of the vertical force at the wheel with time when the vehicle is not using the stabilizer bar is shown in Fig 8. According to this result, the greatest difference between wheels is obtained at the very high-speed state, v_4_ = 100 (km/h). This shows that if the vehicle steers at a higher speed, the change in the vertical force at the wheel will also be more significant. The minimum value of the vertical force at the rear wheel is only F_z21_ = 389.9 (N). This is a very small value. This value warns of a rollover occurring when it drops to zero. If the vehicle uses a mechanical stabilizer bar, the minimum value of the vertical force at the wheel can be further improved, reaching 1202.6 (N), corresponding to the speed of v_4_ (Fig 9). If the active stabilizer bar controlled by the fuzzy 3-inputs algorithm is used, the vehicle’s stability can be greatly improved. According to the results shown in Fig 10, the minimum value of the vertical force can be up to 3209.0 (N). This is a perfectly stable and safe value. It is 8.23 times and 2.67 times larger than the two situations mentioned above. A conclusion can be made that the smaller the difference in dynamic forces between the wheels, the better the stability performance. For the cases where the vehicle travels at lower speeds (a, b, and c), the difference in dynamic forces is most significant when the vehicle does not have the stabilizer bar. Meanwhile, the smallest difference belongs to the situation where the car has the stabilizer bar controlled by a 3-input fuzzy algorithm. These results tend to be similar but differ in their values. These results can be seen more clearly in Tables 3–6.

**Table 3:** Simulation results–Case 1.

	Fuzzy	Mechanical	None
v_1_			
Maximum roll angle	5.32	6.26	6.57
Minimum vertical force	3470.7	1973.3	1356.2
Maximum Roll Index	0.17	0.53	0.68
v_2_			
Maximum roll angle	6.07	7.07	7.41
Minimum vertical force	3376.8	1684.8	991.4
Maximum Roll Index	0.19	0.60	0.76
v_3_			
Maximum roll angle	6.74	7.80	8.15
Minimum vertical force	3290.9	1428.8	670.9
Maximum Roll Index	0.21	0.66	0.84
v_4_			
Maximum roll angle	7.35	8.43	8.80
Minimum vertical force	3209.0	1202.6	389.9
Maximum Roll Index	0.23	0.71	0.91

**Table 4:** Simulation results–Case 2.

	Fuzzy	Mechanical	None
v_1_			
Maximum roll angle	5.91	6.87	7.21
Minimum vertical force	3301.9	1756.2	1077.2
Maximum Roll Index	0.21	0.58	0.74
v_2_			
Maximum roll angle	6.76	7.78	8.15
Minimum vertical force	3184.2	1435.4	670.3
Maximum Roll Index	0.24	0.66	0.84
v_3_			
Maximum roll angle	7.51	8.58	9.00
Minimum vertical force	3066.3	1148.1	296.6
Maximum Roll Index	0.27	0.73	0.93
v_4_			
Maximum roll angle	8.19	9.31	9.38
Minimum vertical force	2953.3	891.1	0
Maximum Roll Index	0.29	0.79	1

**Table 5:** Simulation results–Case 3.

	Fuzzy	Mechanical	None
v_1_			
Maximum roll angle	7.54	8.57	8.98
Minimum vertical force	2218.1	1153.3	314.4
Maximum Roll Index	0.47	0.72	0.92
v_2_			
Maximum roll angle	8.72	9.83	9.13
Minimum vertical force	1941.1	706.3	0
Maximum Roll Index	0.54	0.83	1
v_3_			
Maximum roll angle	9.87	11.05	8.79
Minimum vertical force	1670.3	272.7	0
Maximum Roll Index	0.60	0.94	1
v_4_			
Maximum roll angle	10.95	11.32	8.71
Minimum vertical force	1410.9	0	0
Maximum Roll Index	0.66	1	1

**Table 6:** Simulation results–Case 4.

	Fuzzy	Mechanical	None
v_1_			
Maximum roll angle	7.66	8.69	9.16
Minimum vertical force	1973.9	1112.7	230.4
Maximum Roll Index	0.53	0.73	0.94
v_2_			
Maximum roll angle	8.87	9.96	8.81
Minimum vertical force	1683.3	660.3	0
Maximum Roll Index	0.60	0.84	1
v_3_			
Maximum roll angle	10.06	11.25	9.21
Minimum vertical force	1373.2	198.3	0
Maximum Roll Index	0.67	0.95	1
v_4_			
Maximum roll angle	11.20	11.01	8.80
Minimum vertical force	1051.5	0	0
Maximum Roll Index	0.75	1	1

**Fig 11:**
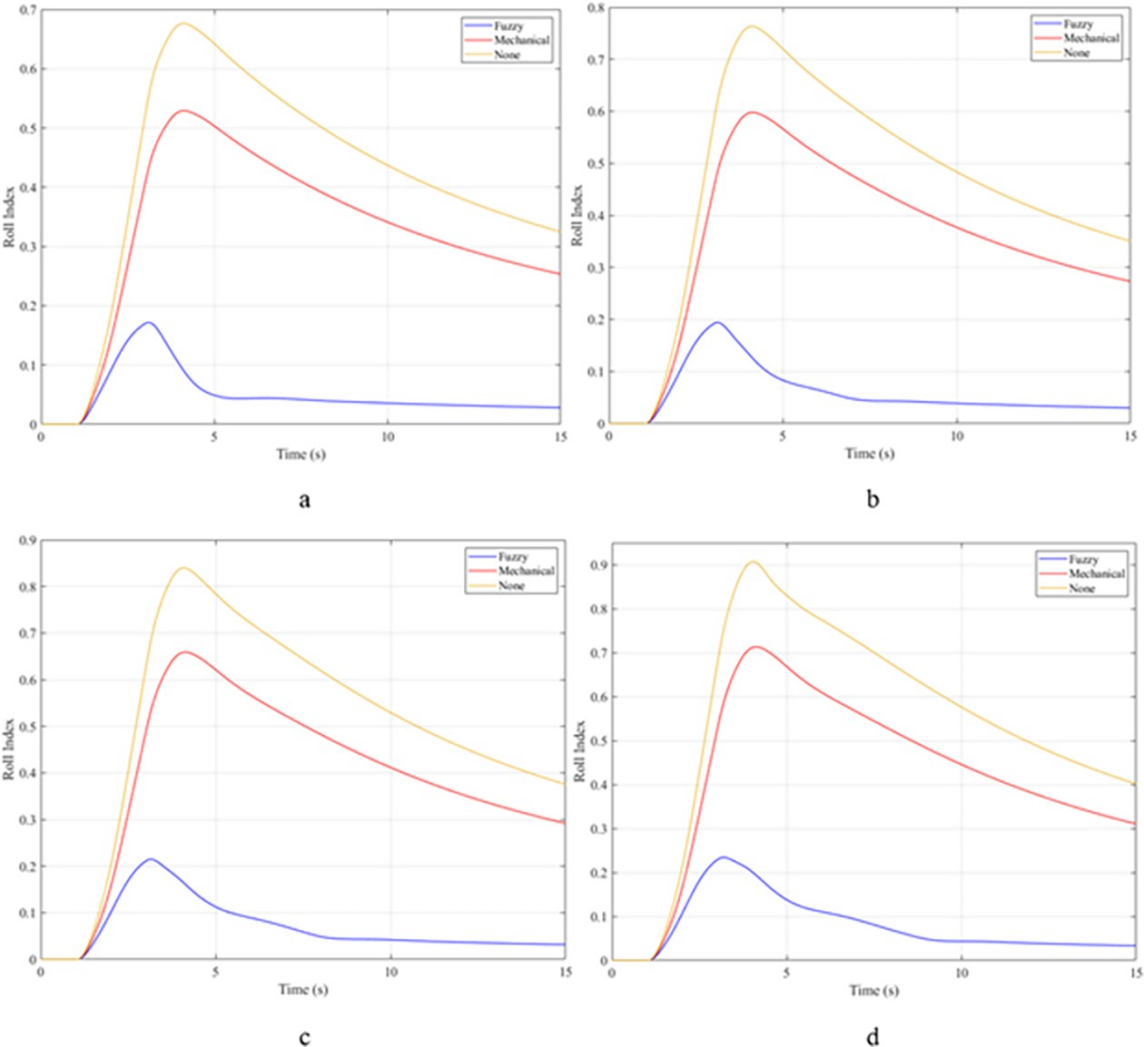
Roll index.

The rollover index (RI) can also be used to determine a vehicle’s hazardous condition. In this article, the rollover index is determined on one axle (front or rear axle). If one wheel of each axle is completely separated from the road surface, that is, the roll index will reach its maximum value, RI = 1. The smaller this value, the greater the safety of the vehicle. The maximum roll index of the vehicle without the stabilizer bar will increase gradually as the vehicle’s speed increases, reaching 0.68; 0.76; 0.84; and 0.91 (Fig 11). The values for the vehicle situation with a passive stabilizer bar are 0.53; 0.60; 0.66; and 0.71, respectively. Meanwhile, the values for the third situation (vehicle with active stabilizer bar) are only 0.17; 0.19; 0.21; and 0.23, corresponding to four-velocity values. According to these results, the stability of the vehicle is always guaranteed when the vehicle uses the stabilizer bar.

#### 3.2.2 Second case

The "J-turn" steering angle used in the first case has not caused any danger to the vehicle when moving. Therefore, it is necessary to use steering angles of greater amplitude. In the second case, a "double lane change" steering angle is used. The amplitude of this steering angle can be up to 8°, which is larger than the first situation. In addition, the use of this steering angle can also help determine the stability of the control system after the steering system returns to its original position.

**Fig 12:**
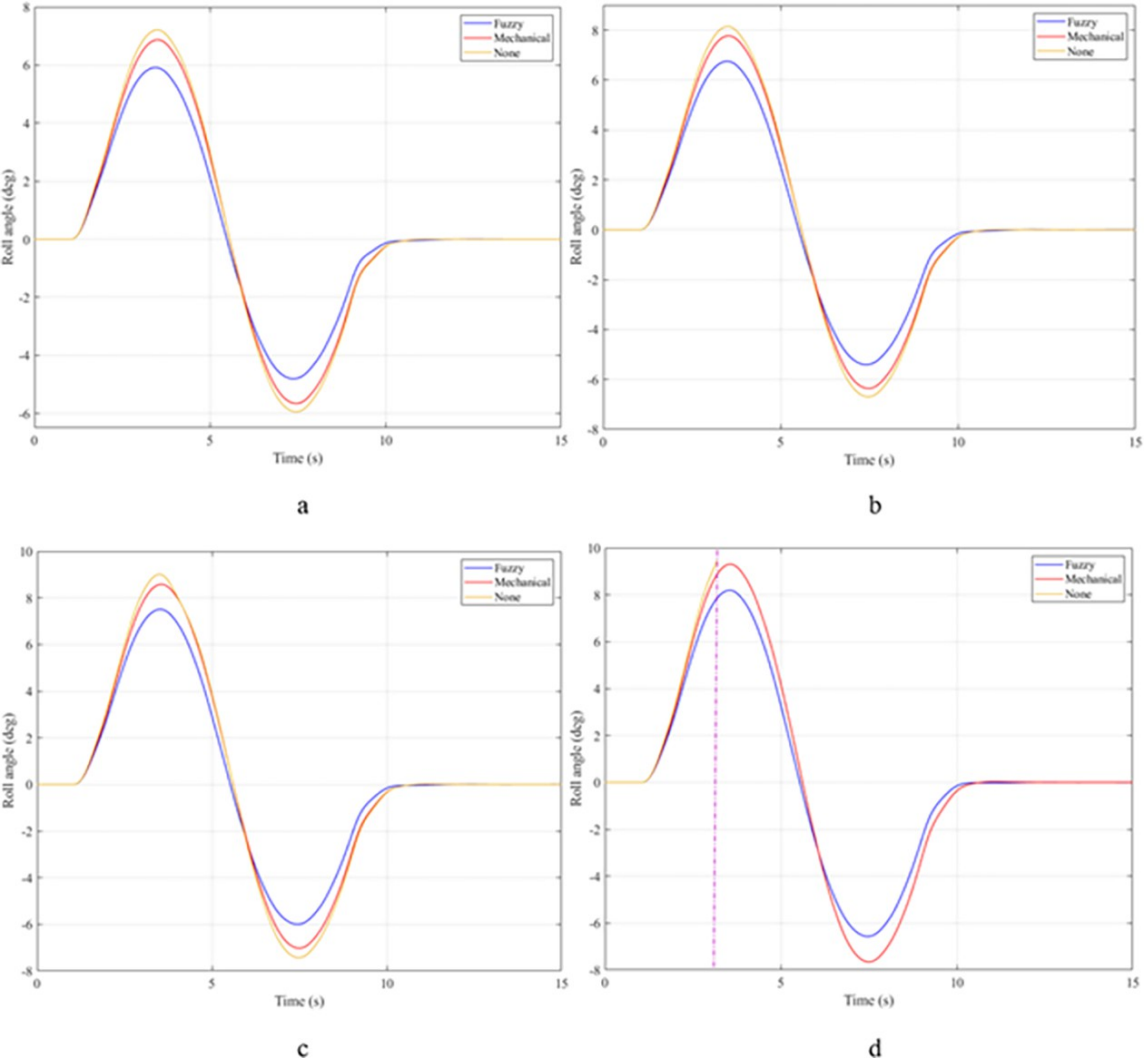
Roll angle.

The roll angle of the vehicle is still a value that is of particular interest when the vehicle is steering. According to the results illustrated in Fig 12, the vehicle’s oscillation consists of two phases. The value of the roll angle in the first phase is greater than that in the second phase, although the magnitude of the steering angle in the two phases is the same. The cause of this phenomenon is the elastic deformation of the tire, which was explained above. At an average speed of v_1_ = 70 (km/h), the maximum value of the roll angle corresponding to all three situations is 5.91°, 6.87°, and 7.21°, respectively. These values are obtained in the first phase of the oscillation. Once the speed increases up to v_2_ = 80 (km/h), these values also increase accordingly, reaching 6.76°, 7.78°, and 8.15°. If the speed continues to increase up to v_3_ = 90 (km/h), the vehicle’s roll angle without using the stabilizer bar has reached 9.00°. While the value of the roll angle when the vehicle uses the hydraulic stabilizer bar is only 7.51°. The difference in values between the situations is quite large. If the vehicle’s speed increases to 100 (km/h), a rollover phenomenon may occur, corresponding to the third situation where the vehicle does not have a stabilizer bar. The limit value of the roll angle in this situation can reach 9.38°.

**Fig 13:**
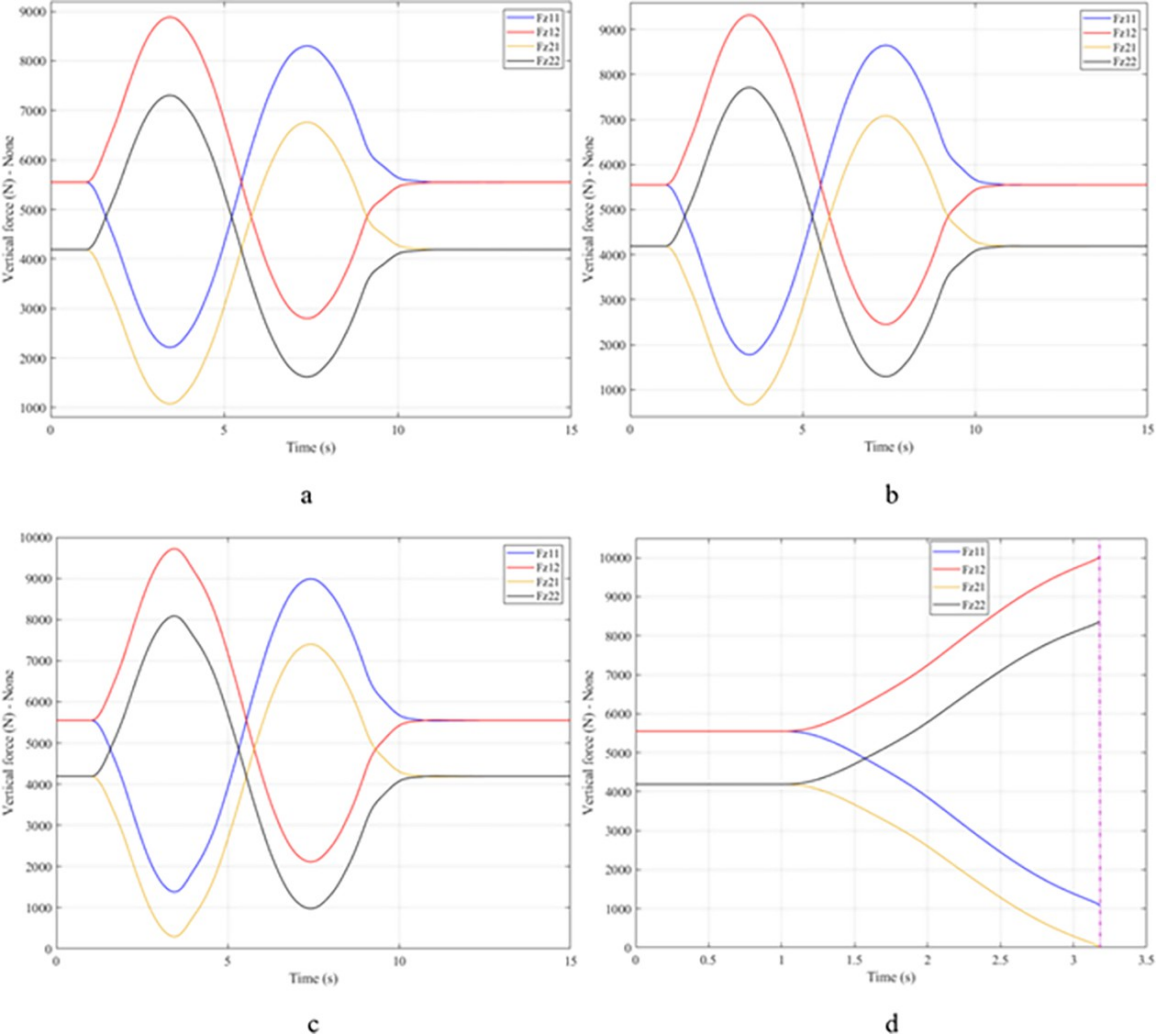
Vertical force–None.

**Fig 14:**
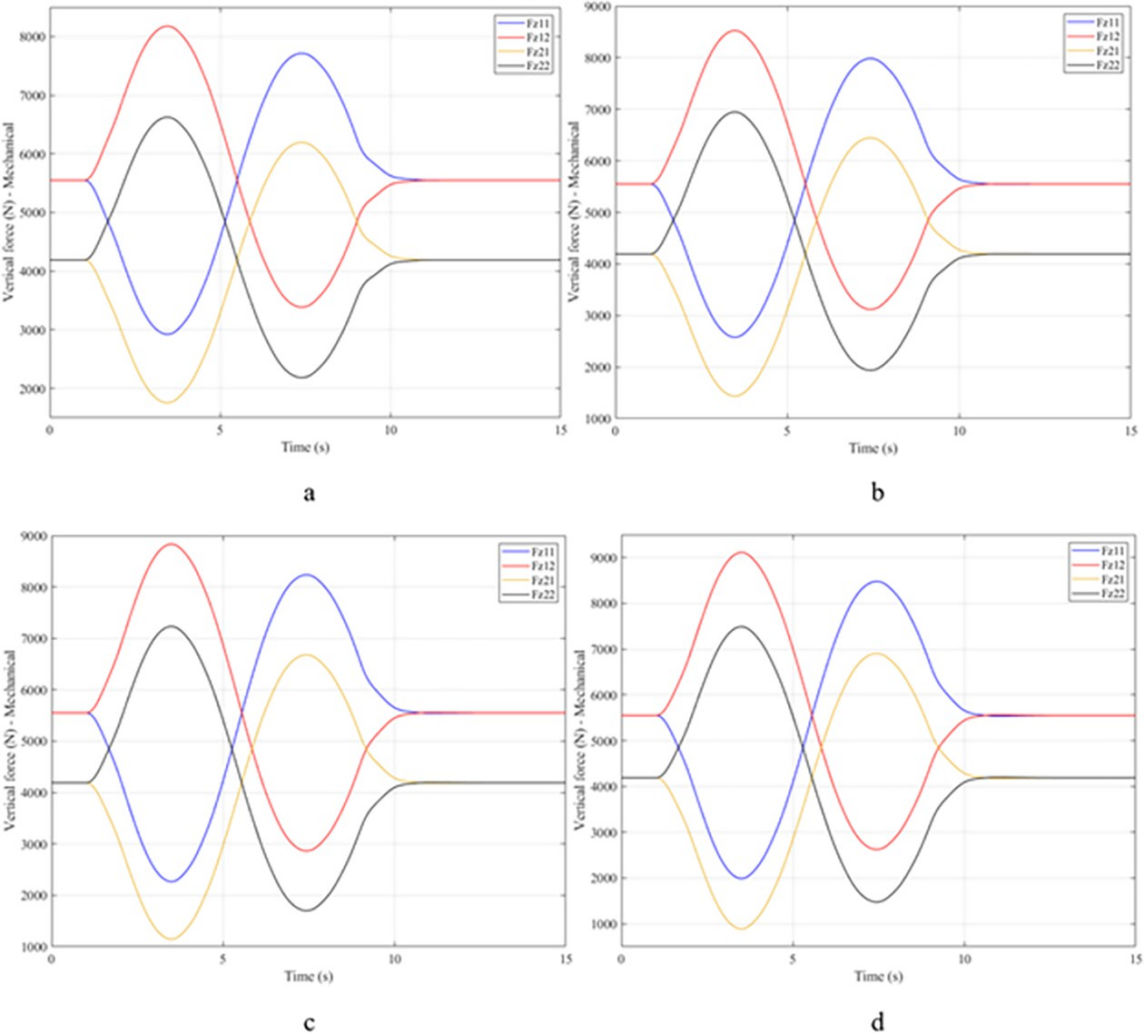
Vertical force–Mechanical.

**Fig 15:**
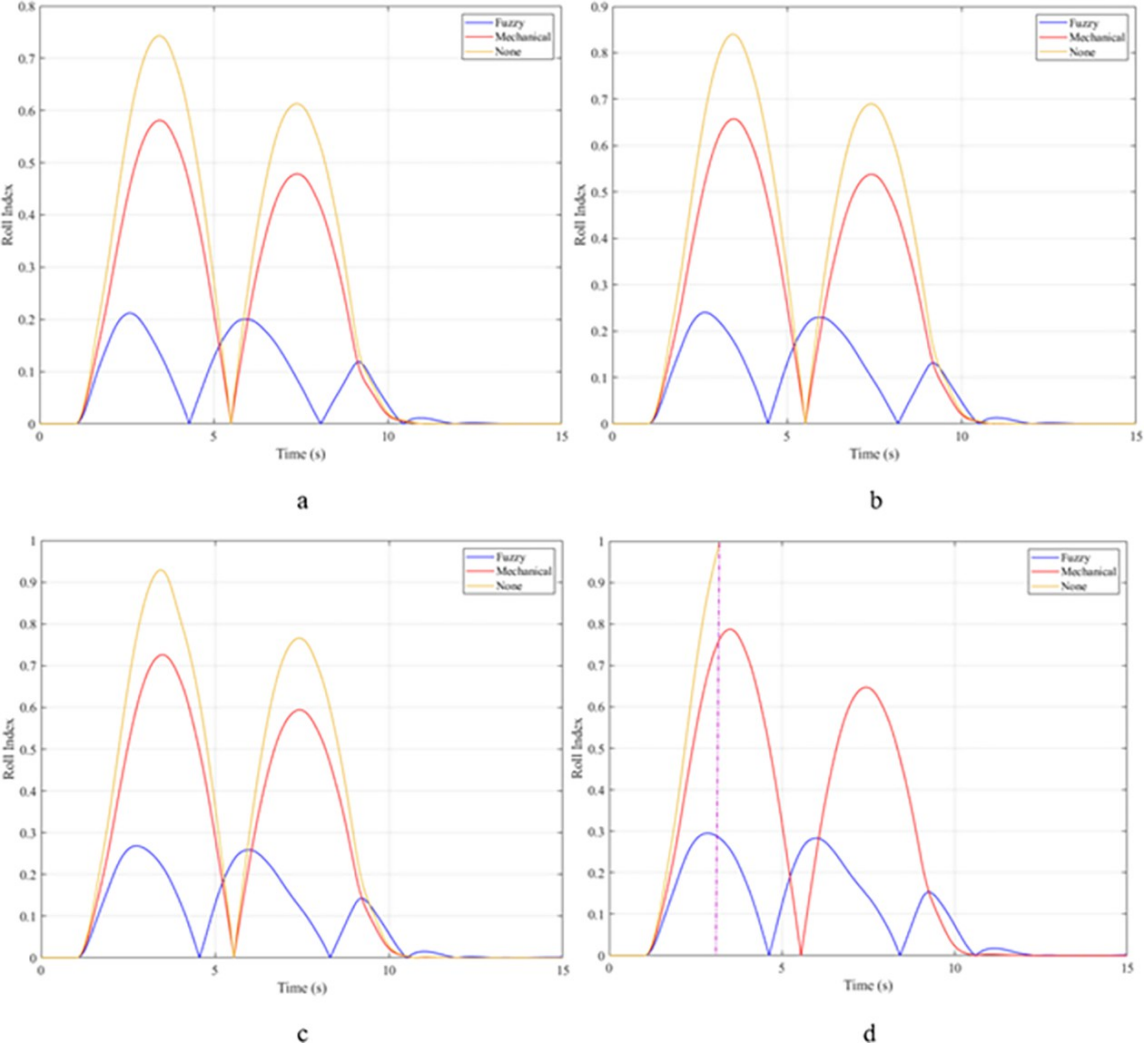
Roll index.

When the vehicle rolls over, i.e., the wheel is completely separated from the road surface, the value of the vertical force at the wheel will be zero (Fig 13), rollover index RI = 1. If the vehicle uses a mechanical stabilizer bar, this value is only 891.1 (N) (Fig 14), corresponding to RI = 0.79 (Fig 15). This is also a rather dangerous limit. If the vehicle speed continues to rise, a rollover phenomenon may also occur even though the vehicle has used the passive stabilizer bar.

**Fig 16:**
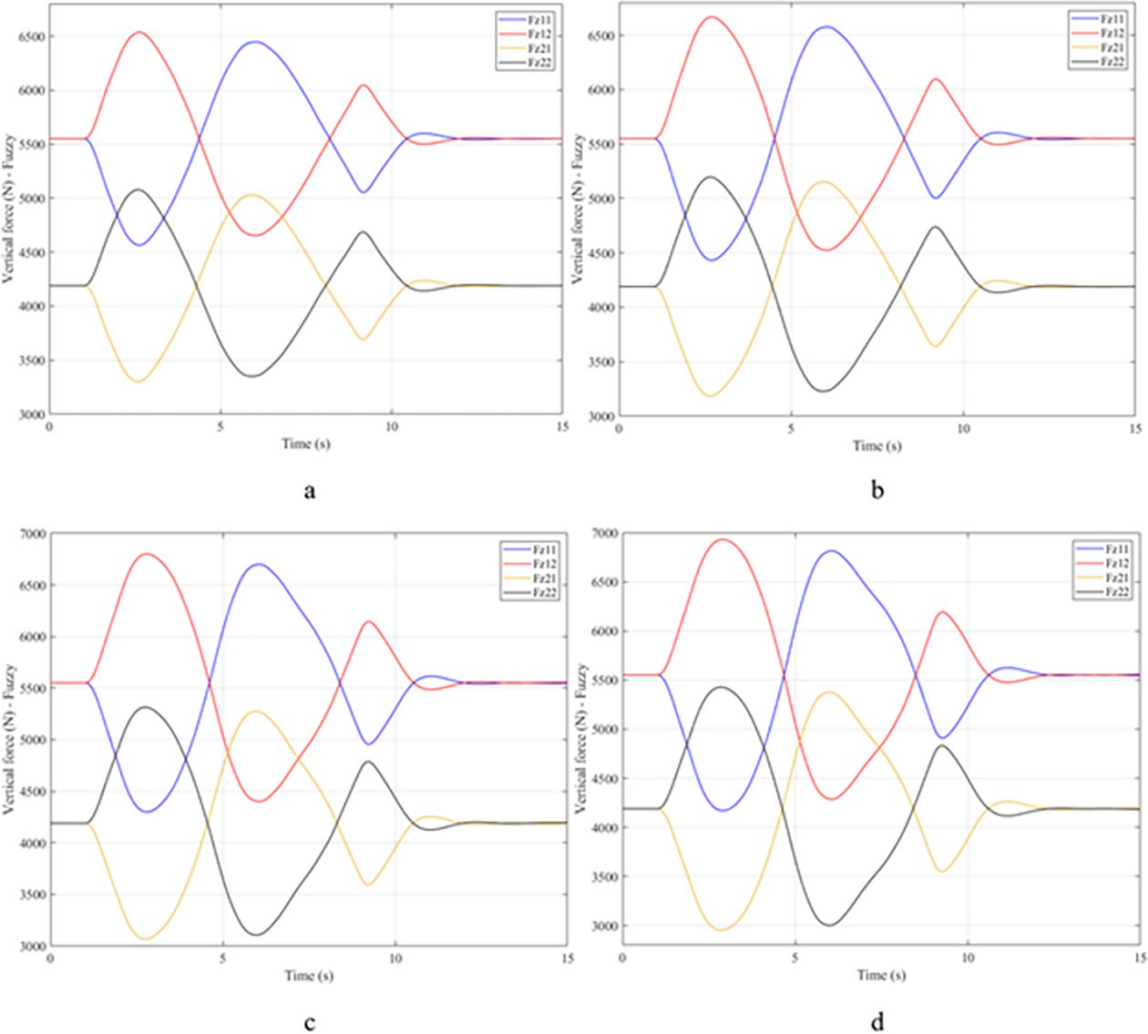
Vertical force–Fuzzy.

Vehicle stability and safety can be ensured once an active stabilizer bar is fitted to the vehicle. At a dangerous speed of v_4_ = 100 (km/h), the minimum value of the vertical force at the rear wheel is 2953.3 (N) (Fig 16). Therefore, its roll index is also quite low, at RI = 0.29. In contrast to the above two situations, the change of vertical force at the wheel in the third situation will consist of three phases. The change in the third phase is smaller than in the previous two phases (Fig 16). The reason for this difference is the design process of a fuzzy three-input algorithm based on the author’s point of view.

For actuator control studies, the stability of the system after the excitation has ended is a very important issue. If the controller is improperly designed, the control signal may continue to be generated even though the input excitations have ceased to exist. This can cause vehicle instability. The fuzzy inputs algorithm used in this article ensures stability in all situations. Once the excitation signal is over, the control signal will also last for a very short time, after which the signal will also cease to exist. This is evidenced by the vertical force difference at the wheel.

#### 3.2.3 Third case

In rollover studies, the "fishhook" steering angle is often used. If the vehicle uses this type of steering angle, instability may occur. Therefore, the performance of the active stabilizer bar can be easily evaluated compared to the passive stabilizer bar.

**Fig 17:**
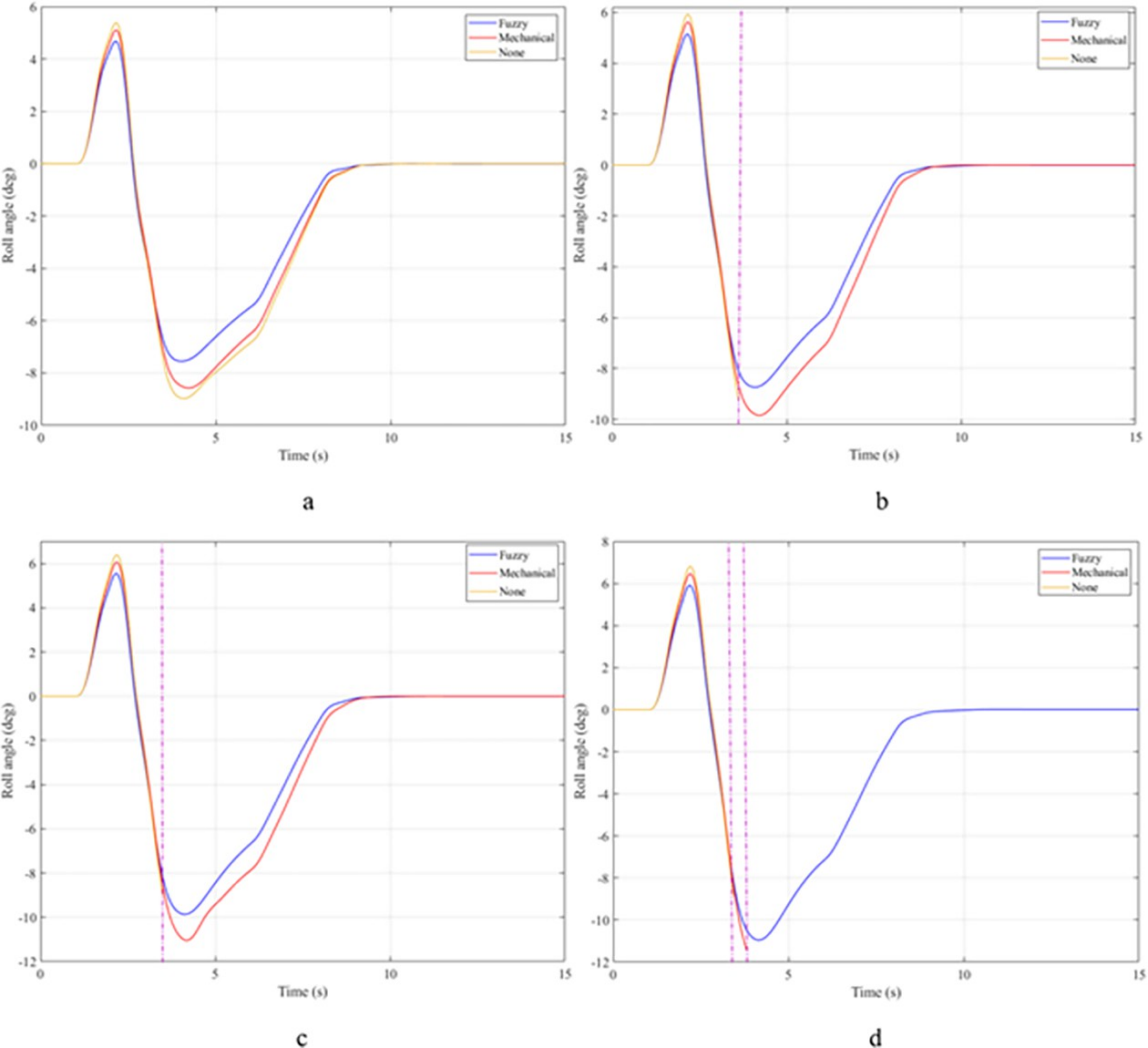
Roll angle.

The "fishhook" steering angle typically involves two phases. The oscillation of the later phase is usually larger than the oscillation of the previous phase. The change of the roll angle with time is shown in Fig 17. According to this result, the rollover would have occurred at v_2_ = 80 (km/h) if the vehicle had not used the stabilizer bar. The limited value of the roll angle reaches 9.13° at the second phase of the oscillation. When the speed increases up to v_3_ = 90 (km/h), the vehicle is more prone to rollover. Because the vehicle rolled over earlier, the limited roll angle is only 8.79°. Meanwhile, the maximum value of the vehicle’s roll angle can be up to 9.87° and 11.05° if the vehicle uses a stabilizer bar. Although these two values are even larger than the limited value of the third situation, the vehicle still does not roll over.

**Fig 18:**
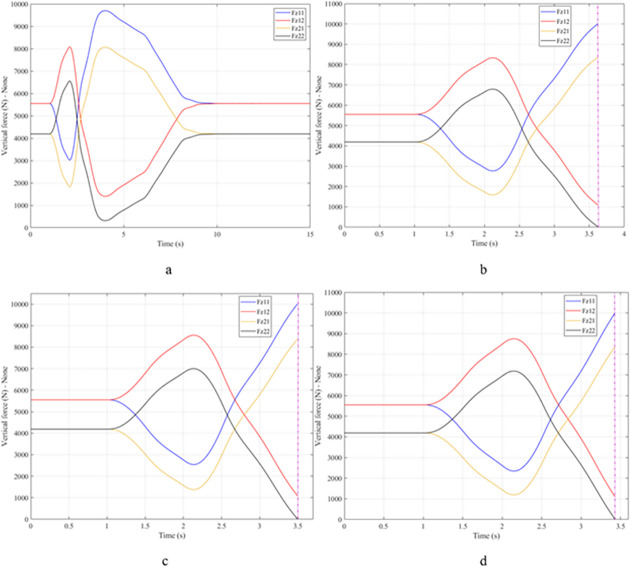
Vertical force–None.

**Fig 19:**
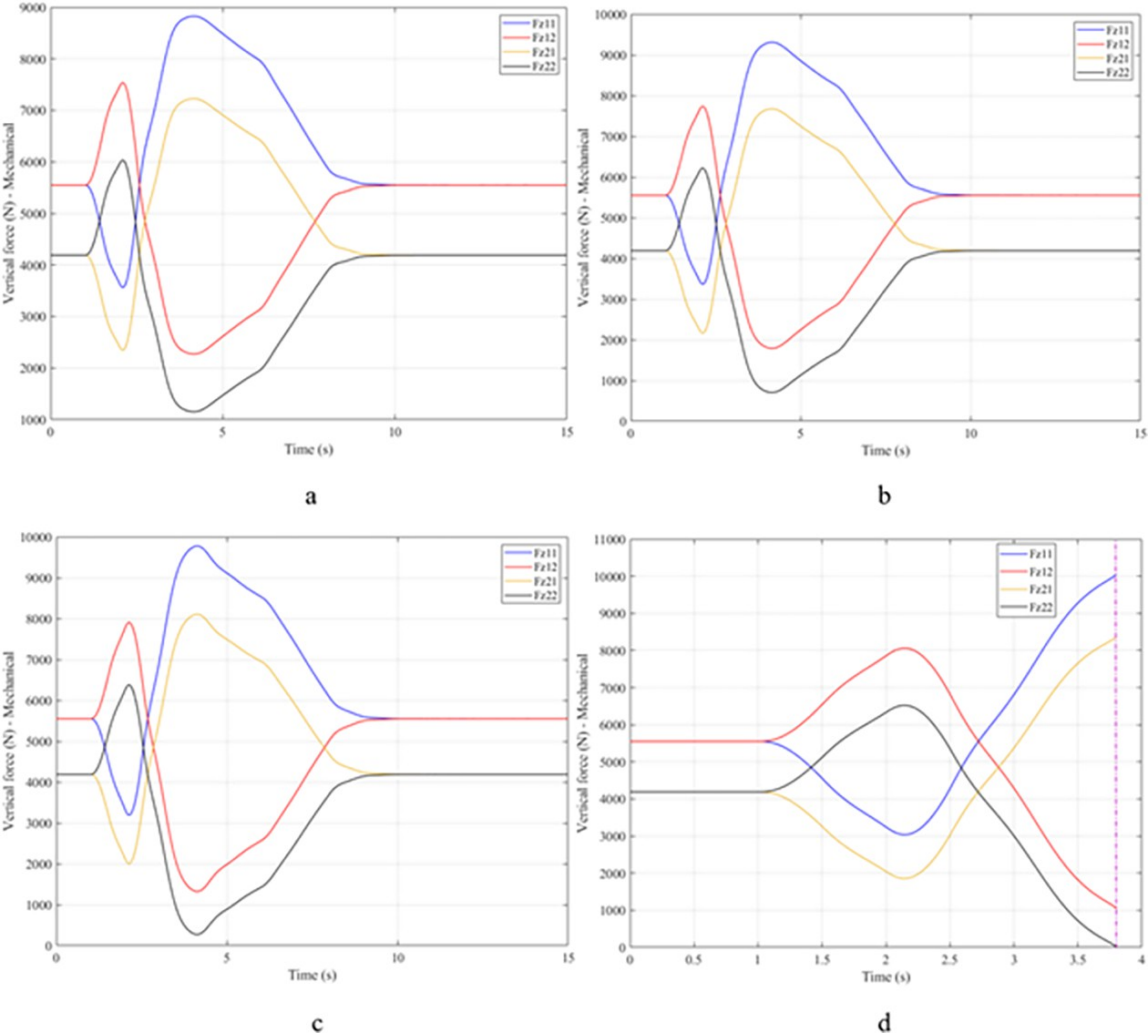
Vertical force–Mechanical.

Once the vehicle’s speed reached its maximum value, v_4_ = 100 (km/h), the rollover phenomenon occurred even when the passive stabilizer bar was used. The limited roll angle of the two situations: the vehicle using the passive stabilizer bar and the vehicle not using the stabilizer bar reached 11.32°, and 8.71° respectively. This result can be understood simply: if the vehicle does not have a stabilizer bar, it will roll over sooner. At v_4_ speed, the rollover phenomenon occurs at time t = 3.4 (s) if the stabilizer bar is not used (Fig 18), and at t = 3.8 (s) if the vehicle is using a mechanical stabilizer bar (Fig 19).

**Fig 20:**
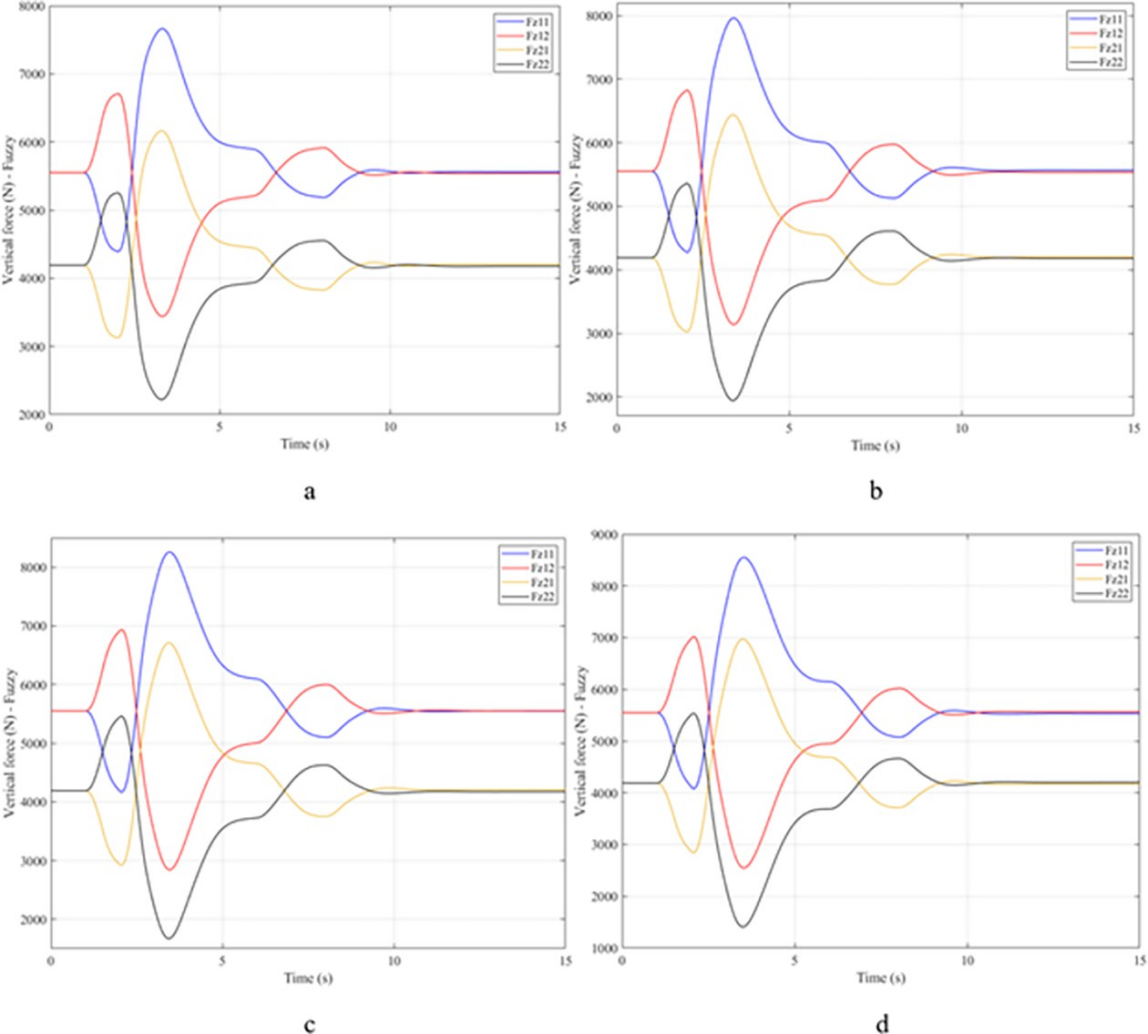
Vertical force–Fuzzy.

**Fig 21:**
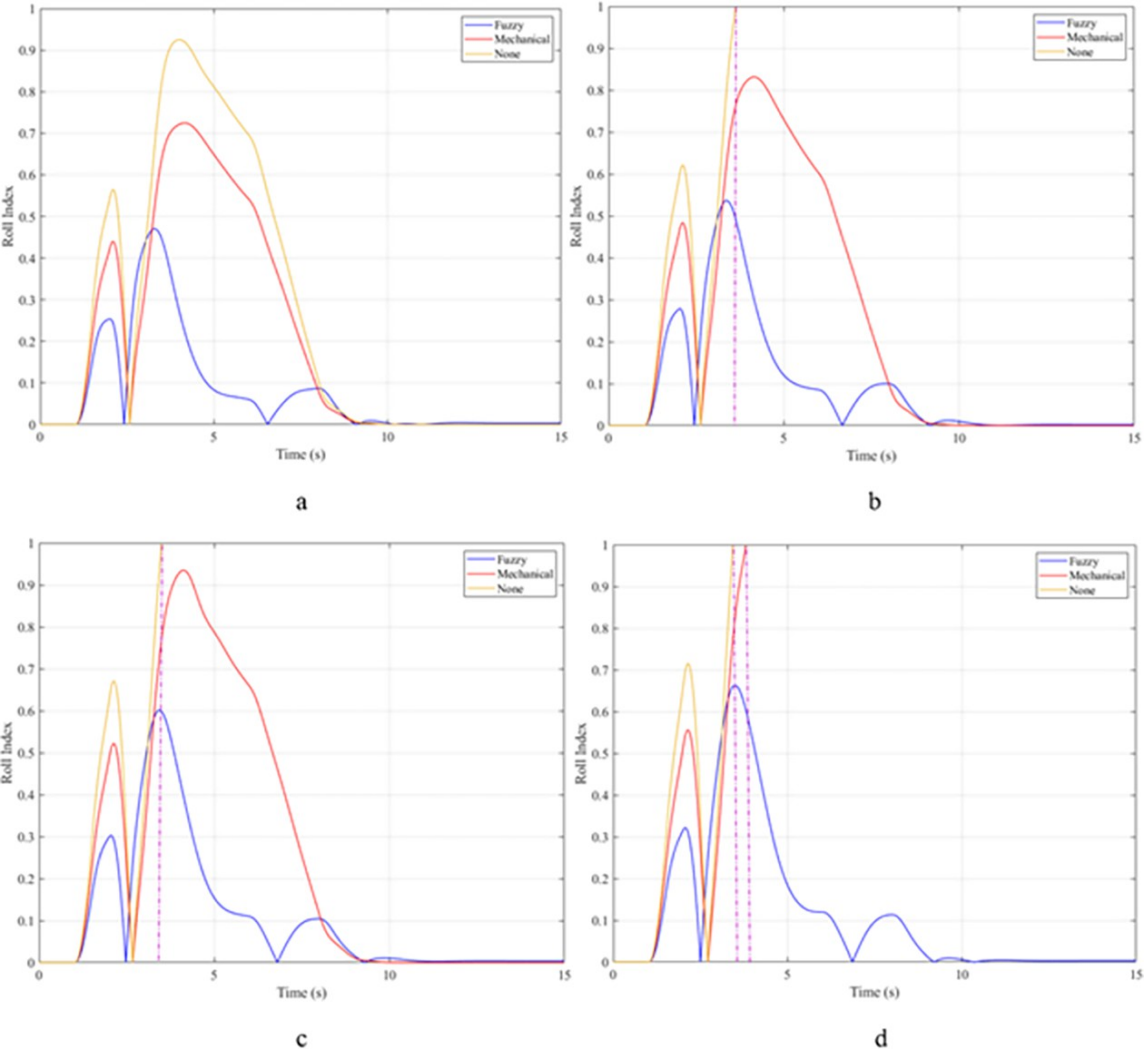
Roll index.

In this case, the stability and safety of the vehicle are still guaranteed if active stabilizer bars are used at both axles of the vehicle. At the maximum speed, v_4_ = 100 (km/h), the minimum value of the vertical force at the wheel is 1410.9 (N) (Fig 20), corresponding to a roll index RI = 0.66 (Fig 21). The hydraulic stabilizer bar has improved the vehicle’s safety in a much more positive way.

#### 3.2.4 Forth case

**Fig 22:**
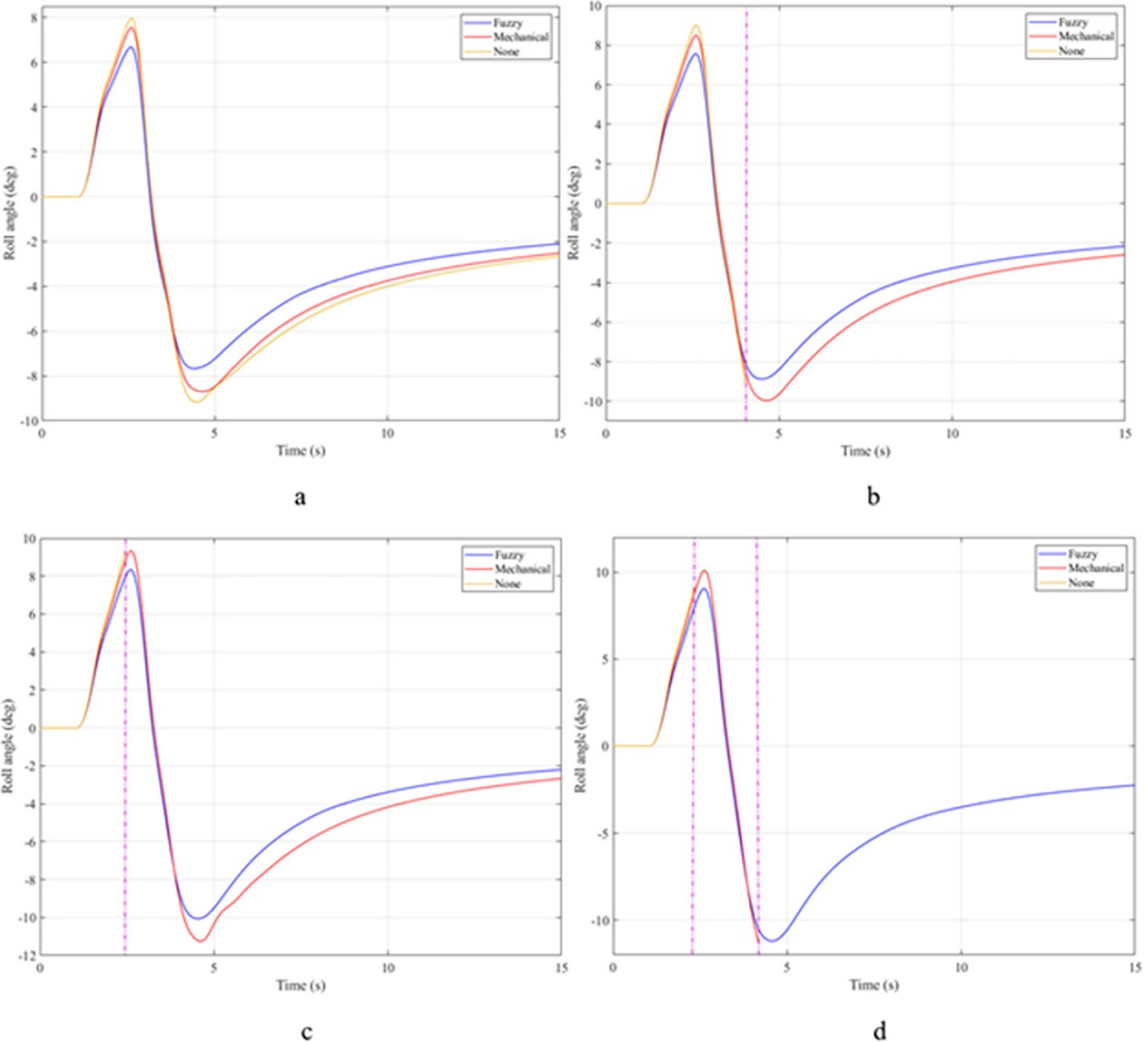
Roll angle.

In the last case, the "fishhook" steering angle is still used. However, the amplitude of the steering angle has increased. This is considered one of the extremely dangerous cases. According to the results shown in Fig 22, the vehicle rolled over as soon as it was moving at v_2_ if the stabilizer bar was rolled at the first phase of the oscillation, with the limited roll angle reaching only 9.21°. At speed v_4_, the vehicle rolled over completely despite being equipped with passive stabilizer bars on both the front and rear axles of the vehicle.

**Fig 23:**
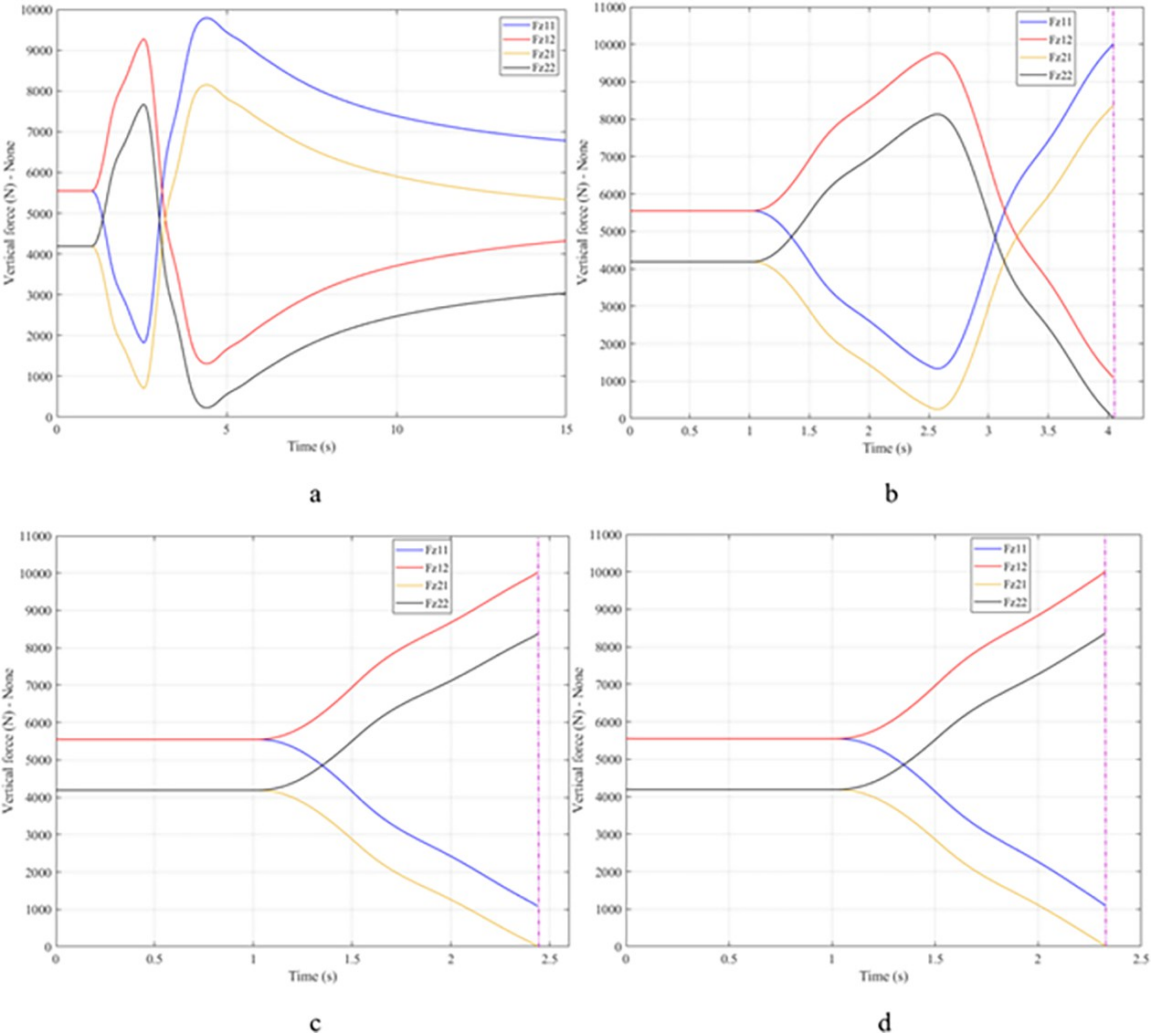
Vertical force–None.

**Fig 24:**
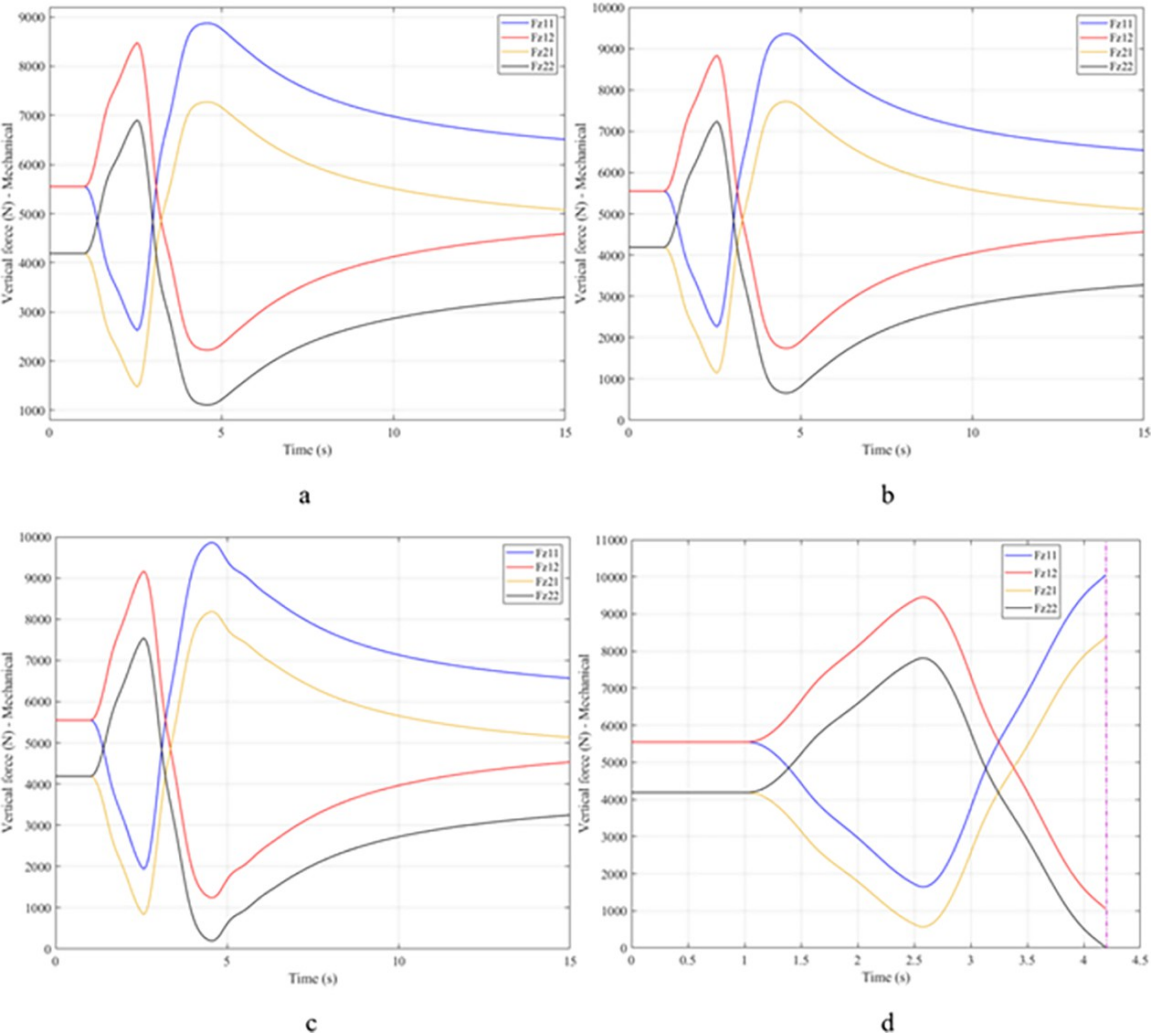
Vertical force–Mechanical.

**Fig 25:**
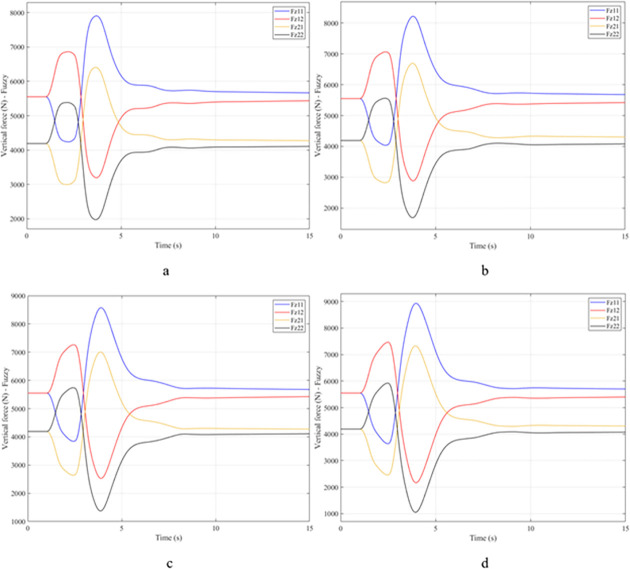
Vertical force–Fuzzy.

**Fig 26:**
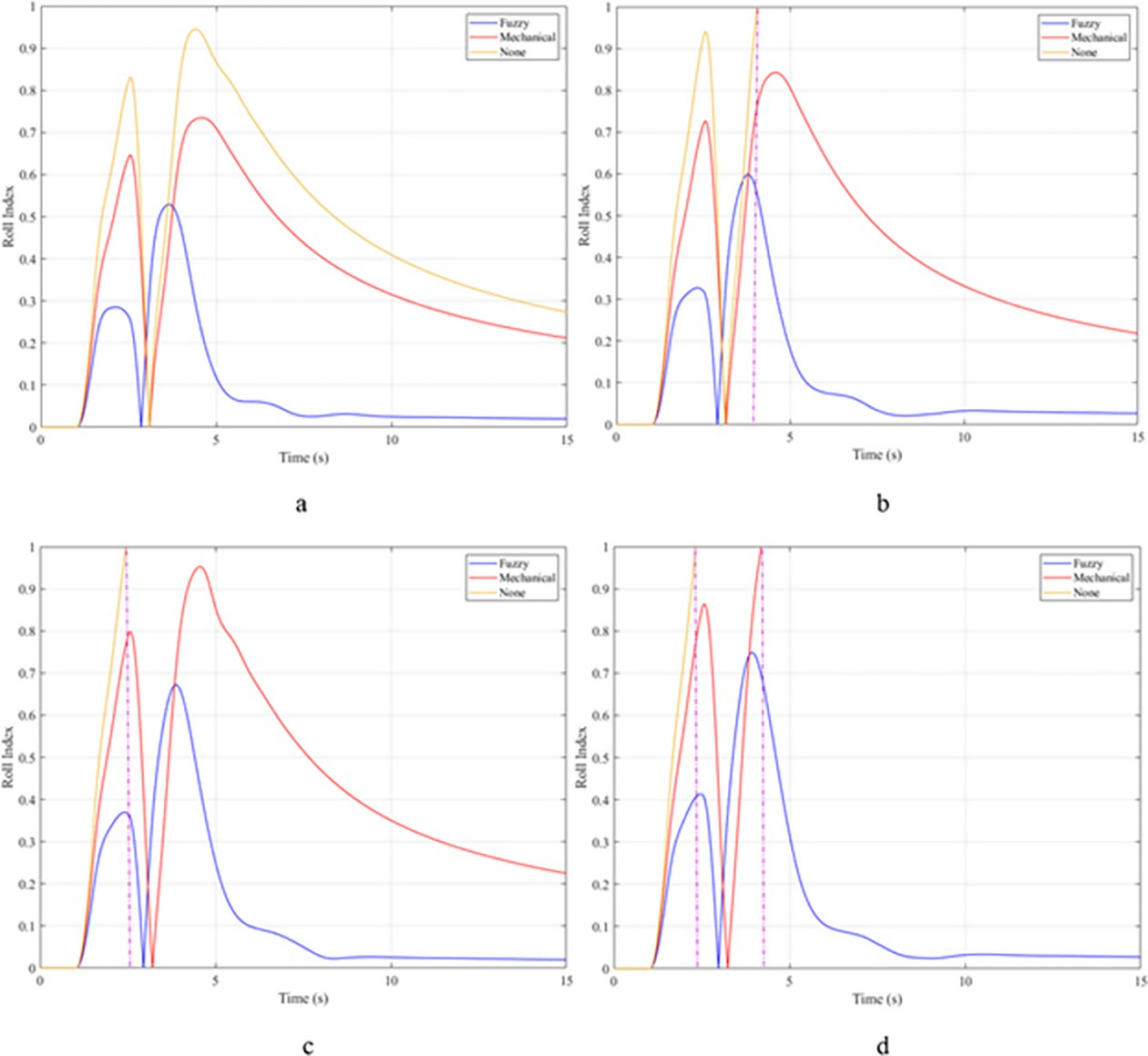
Roll index.

As demonstrated by the graphs in Figs 23 and 24, when the vehicle rolls over, the value of the vertical force at the wheels will be zero. The time when the rollover phenomenon occurs is relative to the situation and is different. If the active stabilizer bar is used to replace the conventional mechanical stabilizer bar, the rollover phenomenon will not occur. At maximum velocity, the minimum value of the vertical force is only 1051.5 (N) (Fig 25). Although this value is not large, however, safety is still guaranteed. The rollover index of the vehicle when using the hydraulic stabilizer bar at speed v_4_ can reach RI = 0.75 (Fig 26). Overall, the effect of using the active stabilizer bar controlled by the fuzzy 3-inputss algorithm is very positive.

The results of the simulation are summarized in Tables 3–6. According to the results from Table 3, the rollover does not occur even when the vehicle is traveling at v_4_. However, this phenomenon occurs in the second case (Table 4) if the vehicle does not have stabilizer bars and the maximum roll angle of the vehicle body reaches 9.38°. In the third case (Table 5), rollover occurs at v_2_, v_3_, and v_4_ if the vehicle is not using the stabilizer bar. Besides, if the car has mechanical stabilizer bars, the rollover occurs only at v_4_ speed (with a maximum roll angle of 11.32°). In the last case (Table 6), the maximum roll angle of the car decreases when the rollover occurs. It can be understood that an automobile can roll over sooner. Once the car is equipped with the hydraulic stabilizer bar controlled by the fuzzy 3-input algorithm, the car rollover will not occur in all investigated cases.

The results obtained from the simulation process help demonstrate the effectiveness of this algorithm. In general, the fuzzy 3-input algorithm brings stability to the system. At the same time, the requirements of the proposed problem are also fully met. Therefore, this new algorithm can be practically applied to today’s cars.

## 4. Conclusions

The phenomenon of vehicle rollover often occurs when traveling at high speed and the driver suddenly steers. Under the influence of centrifugal force, the body will tilt, and the wheels will tend to separate from the road surface. Once the vertical force at the wheel is zero, the vehicle will roll over. The consequences of this phenomenon are extremely dangerous. The use of a stabilizer bar to limit the rollover phenomenon is a very suitable solution. This article focuses on establishing the model of a complex dynamic to describe vehicle oscillations. These complex dynamics model is a combination of the model of spatial dynamics (7 DOF), the nonlinear double-track dynamics model (3 DOF), and the nonlinear tire model. Besides, a fuzzy three-input control algorithm is proposed to control the active stabilizer bar’s operation. The simulation process is performed by MATLAB software with four specific cases. In each case, the vehicle’s speed will be increased from 70 (km/h) to 100 (km/h). There are three situations simulated in each case, including a vehicle without a stabilizer bar, a vehicle using a passive stabilizer bar, and a vehicle using an active stabilizer bar. With input data such as steering angle and speed of movement, the output data of the simulation problem includes roll angle, vertical force at the wheels, and roll index.

The results of the research showed the outstanding performance of the active stabilizer bar controlled by the fuzzy 3-inputs algorithm. The values of the roll angle, the vertical force difference, and the roll index of the vehicle are all significantly reduced when the hydraulic stabilizer bar is used. In all cases, the stability and safety of the vehicle are always ensured when the vehicle uses the active stabilizer bar. Conversely, a rollover can occur at high speeds if the vehicle does not have a stabilizer bar or if the vehicle uses only a conventional passive stabilizer bar. The control signal of the controller follows the excitation signal very well. Once the excitation signal ends, the control signal also terminates. So, the performance of the controller is very high.

This research only performed the simulation process. The dynamics model and control algorithm used in this article are quite complex. In the future, several experiments can be carried out to demonstrate the effectiveness of the active stabilizer bar with the fuzzy three-input control algorithm.

## Supporting information

S1 File (ZIP)

## List of abbreviations

ϕ: Roll angle, deg
ψ: Yaw angle, deg
θ: Pitch angle, deg
β: Heading angle, deg
δ: Steering angle, deg
α: Slip angle, deg
a_y_: Lateral acceleration, m/s^2^
F_AHSB_: Force of the active hydraulic stabilizer bar, N
F_MSB_: Force of the mechanical stabilizer bar, N
F_C_: Force of the damper, N
F_K_: Force of the spring, N
F_KT_: Force of the tire, N
F_x_: Longitudinal force of the wheel, N
F_y_: Lateral force of the wheel, N
F_z_: Vertical force of the wheel, N
g: Gravitational acceleration, m/s^2^
m: Sprung mass (vehicle body), kg
m_ij_: Unsprung mass, kg
M_z_: Moment of the tire, Nm
h_x_: Distance from center of gravity to roll axis, m
h_y_: Distance from center of gravity to pitch axis, m
t_w_: Half of the track width, m
J_x, y, z_: Moment of inertia of the x, y, z-axis, kgm^2^
s: Slip ratio
v_x_: Longitudinal velocity, m/s
v_y_: Lateral velocity, m/s
z: Displacement of the sprung mass, m
z_ij_: Displacement of the unsprung mass, m
