# Supplementary material for: Using a novel fuzzy 3-inputs algorithms to control the active hydraulic stabilizer bar with the complex model of the vehicle nonlinear dynamics
Source: PLoS One. 2023 Mar 7;18(3):e0282505. doi: 10.1371/journal.pone.0282505 (PMC9990926; doi:10.1371/journal.pone.0282505)
Supplement: S1 File — (ZIP) [file pone.0282505.s001.zip › RESULTS.docx]

**RESULTS**

Case 1:

|  | Fuzzy | Mechanical | None |
| --- | --- | --- | --- |
| v_1_ | | | |
| Maximum roll angle | 5.32 | 6.26 | 6.57 |
| Minimum vertical force | 3470.7 | 1973.3 | 1356.2 |
| Maximum Roll Index | 0.17 | 0.53 | 0.68 |
| v_2_ | | | |
| Maximum roll angle | 6.07 | 7.07 | 7.41 |
| Minimum vertical force | 3376.8 | 1684.8 | 991.4 |
| Maximum Roll Index | 0.19 | 0.60 | 0.76 |
| v_3_ | | | |
| Maximum roll angle | 6.74 | 7.80 | 8.15 |
| Minimum vertical force | 3290.9 | 1428.8 | 670.9 |
| Maximum Roll Index | 0.21 | 0.66 | 0.84 |
| v_4_ | | | |
| Maximum roll angle | 7.35 | 8.43 | 8.80 |
| Minimum vertical force | 3209.0 | 1202.6 | 389.9 |
| Maximum Roll Index | 0.23 | 0.71 | 0.91 |

Case 2:

|  | Fuzzy | Mechanical | None |
| --- | --- | --- | --- |
| v_1_ | | | |
| Maximum roll angle | 5.91 | 6.87 | 7.21 |
| Minimum vertical force | 3301.9 | 1756.2 | 1077.2 |
| Maximum Roll Index | 0.21 | 0.58 | 0.74 |
| v_2_ | | | |
| Maximum roll angle | 6.76 | 7.78 | 8.15 |
| Minimum vertical force | 3184.2 | 1435.4 | 670.3 |
| Maximum Roll Index | 0.24 | 0.66 | 0.84 |
| v_3_ | | | |
| Maximum roll angle | 7.51 | 8.58 | 9.00 |
| Minimum vertical force | 3066.3 | 1148.1 | 296.6 |
| Maximum Roll Index | 0.27 | 0.73 | 0.93 |
| v_4_ | | | |
| Maximum roll angle | 8.19 | 9.31 | 9.38 |
| Minimum vertical force | 2953.3 | 891.1 | 0 |
| Maximum Roll Index | 0.29 | 0.79 | 1 |

Case 3:

|  | Fuzzy | Mechanical | None |
| --- | --- | --- | --- |
| v_1_ | | | |
| Maximum roll angle | 7.54 | 8.57 | 8.98 |
| Minimum vertical force | 2218.1 | 1153.3 | 314.4 |
| Maximum Roll Index | 0.47 | 0.72 | 0.92 |
| v_2_ | | | |
| Maximum roll angle | 8.72 | 9.83 | 9.13 |
| Minimum vertical force | 1941.1 | 706.3 | 0 |
| Maximum Roll Index | 0.54 | 0.83 | 1 |
| v_3_ | | | |
| Maximum roll angle | 9.87 | 11.05 | 8.79 |
| Minimum vertical force | 1670.3 | 272.7 | 0 |
| Maximum Roll Index | 0.60 | 0.94 | 1 |
| v_4_ | | | |
| Maximum roll angle | 10.95 | 11.32 | 8.71 |
| Minimum vertical force | 1410.9 | 0 | 0 |
| Maximum Roll Index | 0.66 | 1 | 1 |

Case 4:

|  | Fuzzy | Mechanical | None |
| --- | --- | --- | --- |
| v_1_ | | | |
| Maximum roll angle | 7.66 | 8.69 | 9.16 |
| Minimum vertical force | 1973.9 | 1112.7 | 230.4 |
| Maximum Roll Index | 0.53 | 0.73 | 0.94 |
| v_2_ | | | |
| Maximum roll angle | 8.87 | 9.96 | 8.81 |
| Minimum vertical force | 1683.3 | 660.3 | 0 |
| Maximum Roll Index | 0.60 | 0.84 | 1 |
| v_3_ | | | |
| Maximum roll angle | 10.06 | 11.25 | 9.21 |
| Minimum vertical force | 1373.2 | 198.3 | 0 |
| Maximum Roll Index | 0.67 | 0.95 | 1 |
| v_4_ | | | |
| Maximum roll angle | 11.20 | 11.01 | 8.80 |
| Minimum vertical force | 1051.5 | 0 | 0 |
| Maximum Roll Index | 0.75 | 1 | 1 |
